# Aggregated Tau activates NLRP3–ASC inflammasome exacerbating exogenously seeded and non-exogenously seeded Tau pathology in vivo

**DOI:** 10.1007/s00401-018-01957-y

**Published:** 2019-02-05

**Authors:** Ilie-Cosmin Stancu, Niels Cremers, Hannah Vanrusselt, Julien Couturier, Alexandre Vanoosthuyse, Sofie Kessels, Chritica Lodder, Bert Brône, François Huaux, Jean-Noël Octave, Dick Terwel, Ilse Dewachter

**Affiliations:** 1 Biomedical Research Institute, Hasselt University, 3500 Hasselt, Belgium; 2 Alzheimer Dementia Group, Institute of Neuroscience, Catholic University of Louvain, 1200, Brussels, Belgium; 3 Louvain Center for Toxicology and Applied Pharmacology, Catholic University of Louvain, 1200, Brussels, Belgium

**Keywords:** AD and Tauopathies, Tau, Tau pathology, Propagation of Tau pathology, In vivo models, Inflammation

## Abstract

Brains of Alzheimer’s disease patients are characterized by the presence of amyloid plaques and neurofibrillary tangles, both invariably associated with neuroinflammation. A crucial role for NLRP3–ASC inflammasome [NACHT, LRR and PYD domains-containing protein 3 (NLRP3)–Apoptosis-associated speck-like protein containing a CARD (ASC)] in amyloid-beta (Aβ)-induced microgliosis and Aβ pathology has been unequivocally identified. Aβ aggregates activate NLRP3–ASC inflammasome (Halle et al. in Nat Immunol 9:857–865, 2008) and conversely NLRP3–ASC inflammasome activation exacerbates amyloid pathology in vivo (Heneka et al. in Nature 493:674–678, 2013), including by prion-like ASC-speck cross-seeding (Venegas et al. in Nature 552:355–361, 2017). However, the link between inflammasome activation, as crucial sensor of innate immunity, and Tau remains unexplored. Here, we analyzed whether Tau aggregates acting as prion-like Tau seeds can activate NLRP3–ASC inflammasome. We demonstrate that Tau seeds activate NLRP3–ASC-dependent inflammasome in primary microglia, following microglial uptake and lysosomal sorting of Tau seeds. Next, we analyzed the role of inflammasome activation in prion-like or templated seeding of Tau pathology and found significant inhibition of exogenously seeded Tau pathology by ASC deficiency in Tau transgenic mice. We furthermore demonstrate that chronic intracerebral administration of the NLRP3 inhibitor, MCC950, inhibits exogenously seeded Tau pathology. Finally, ASC deficiency also decreased non-exogenously seeded Tau pathology in Tau transgenic mice. Overall our findings demonstrate that Tau-seeding competent, aggregated Tau activates the ASC inflammasome through the NLRP3–ASC axis, and we demonstrate an exacerbating role of the NLRP3–ASC axis on exogenously and non-exogenously seeded Tau pathology in Tau mice in vivo. The NLRP3–ASC inflammasome, which is an important sensor of innate immunity and intensively explored for its role in health and disease, hence presents as an interesting therapeutic approach to target three crucial pathogenetic processes in AD, including prion-like seeding of Tau pathology, Aβ pathology and neuroinflammation.

**Electronic supplementary material:**

The online version of this article (10.1007/s00401-018-01957-y) contains supplementary material, which is available to authorized users.

## Introduction

Brains of Alzheimer’s disease patients are characterized by the presence of amyloid plaques and neurofibrillary tangles, respectively composed of Aβ peptides and hyperphosphorylated Tau, and both invariably associated with neuroinflammatory changes [85, 86]. Genetic evidence, biomodelling data and biomarker analysis support an initiating role of accumulating Aβ in the pathogenetic process in EOFAD and sporadic AD patients [39, 47, 48]. While accumulating Aβ presents as a key initiator, but difficult disease-modifying target, there is a growing focus on multi-targeted strategies aiming at including key pathogenetic targets downstream of Aβ, which include Tau [11, 57] and its prion-like or templated propagation and neuroinflammatory changes [40, 64, 80, 99] as key pathogenetic processes in AD.

An executive role of Tau in the pathogenetic process of AD and related Tauopathies is substantiated by (i) the existence of a family of neurodegenerative disorders all characterized by Tau-aggregation, by (ii) the identification of MAPT mutations autosomal dominantly linked to these Tauopathies, indicating that Tau dysfunction causes neurodegeneration, and by (iii) the strong correlation of progression of Tau pathology with progression of disease symptoms in AD—generally considered as a secondary Tauopathy [9, 11, 85, 92]. Furthermore, (iv) prion-like or templated propagation of Tau pathology has been consistently and reproducibly shown in in vitro and in vivo models, highlighting a self-propagating effect of Tau pathology once initiated [3, 12, 24–26, 30–33, 36, 37, 44, 49, 50, 56, 62, 77, 84, 89, 96, 97]. Prion-like propagation of Tau pathology thereby presents as a compelling mechanism for the progressive and characteristic development of Tau pathology, remarkably strong correlated with symptom progression in AD. Importantly, the presence of Tau seeds has been demonstrated not only in brains of Tau transgenic mice, but also in brains of patients [26, 45, 52] using a sensitive Tau seeding detector assay. This not only highlights the relevance of this process in human AD brains [75] but also emphasizes the interest to identify mechanisms to inhibit or target templated propagation of Tau pathology. Hence, the molecular and cellular processes involved in seeding and spreading of Tau pathology and their modulators present a topic of high interest, and represent highly interesting therapeutic targets.

Brains of AD patients and patients with Tauopathies are invariably characterized by neuroinflammatory changes, raising a keen interest in their contribution to the pathogenetic process. Neuroinflammation and innate immunity, in particular, are hence increasingly investigated for their translational potential in neurodegenerative disorders including AD [40, 41, 74, 80]. Innate immunity and inflammasome are crucial in health and disease and, therefore, very intensively investigated [40, 41, 59–61, 66, 80]. The inflammasomes are the sensors of the innate immune system and induce inflammation in response to infectious ‘attacks’ [35] recognized by danger-associated signals [pathogen-associated molecular patterns (PAMPS) and danger-associated molecular patterns (DAMPS)] [40, 41, 59–61, 66, 80]. Inflammasome becomes activated by a dual stimuli leading to the activation of pattern recognition receptors (PRRs), including NOD-like receptors or NLRs. The NLRP3 inflammasome can be activated by structurally diverse stimuli including ATP, imidazoquinoline derivatives, various crystals as well as by bacterial toxins and also Aβ peptides displaying an amyloid structure [21, 51, 70, 72, 76]. NLRP3 activation induces heteromer formation or aggregation of ASC, leading to caspase-1 activation [35], which subsequently can cleave pro-interleukin-1 beta (pro-IL-1β) to active IL-1β. NLRP3–ASC inflammasome activation induces an increase in cytokine and chemokine concentrations and microglial activation, while its activation can also induce microglial death through pyroptosis [43]. Elegant work demonstrated that aggregated Aβ displaying amyloid structure [38, 55] activates the NLRP3–ASC inflammasome, following endo-lysosomal uptake and damage [38], resulting in the activation of caspase-1, subsequent cleavage of pro-IL-1β to IL-1β, and microglial activation [38]. NLRP3 inflammasome assembly was subsequently demonstrated to actively contribute to amyloid pathology [42, 95]. Heneka et al. elegantly demonstrated that inflammasome inhibition through NLRP3 or caspase-1 deficiency inhibits amyloid pathology in APP/PS1 transgenic mice [42, 95], with alterations in microglial phenotypes and phagocytosis capacity as potential contributing mechanisms. These findings were further confirmed using pharmacological NLRP3 inhibitors [16, 19]. Interestingly, ASC specks—fibrillar ASC aggregates formed upon inflammasome activation—have been shown to be released following inflammasome activation and to be taken up by ‘receptor’ microglial cells thereby contributing to the prion-like propagation of inflammasome activation [10, 23] and microglial activation in a prion-like way. This is particularly interesting in the context of prion-like propagation in AD and related neurodegenerative disorders. Venegas et al. demonstrated that aggregation of ASC into prion-like ASC specks exacerbated amyloid pathology [95], by a cross-seeding mechanism. While the relation between Aβ and inflammasome has been analyzed in exquisite detail, the relation between inflammasome and Tau and prion-like propagation of Tau pathology remains unexplored.

Neuropathological and imaging studies have revealed microglial changes associated with Tau pathology in brains of primary Tauopathy patients and animal models recapitulating Tau pathology. It must be noted that neuroinflammatory changes are a prominent characteristic of Tauopathies per se, also in the absence of amyloid plaque pathology, highlighting the association between Tau pathology and microglial changes [5, 18, 46, 64, 86, 90, 100]. This has been demonstrated in postmortem brain tissue [5, 46, 90, 100] as well as using in vivo PET imaging in Tauopathy patients [27, 28, 33]. Neuroinflammatory alterations associated with Tau pathology not only include altered chemokine and cytokine profiles and microglial activation, but also microglial degeneration [5, 18, 46, 64, 86, 90, 100]. While detailed analysis of Tau-associated neuroinflammatory processes will yield insight into their association with different stages of the disease process, these changes are in line with a potential role for inflammasome activation by Tau. In vivo animal models have furthermore demonstrated an active role of microglial activation and IL-1β in the pathogenetic process of Tauopathies [6, 101], and indicated microgliosis associated with early or soluble Tau aggregates and preceding the development of full-blown mature neurofibrillary tangles (NFTs) [26, 45, 53, 78, 101]. As soluble Tau aggregates and Tau seeds display an “amyloid” or aggregated structure similar to Aβ [13, 22, 34, 54, 55], we hypothesized that Tau seeds could activate the NLRP3–ASC inflammasome and thereby contribute to exogenously and non-exogenously seeded Tau pathology in mice in vivo. We here hence focus on the role of microglial activation, and particularly ASC-dependent inflammasome activation in the pathogenetic process of Tauopathies focusing on its effect on exogenously seeded and non-exogenously seeded Tau pathology.

In this work, we, therefore, analyzed whether Tau aggregates, which display similar amyloid structure as Aβ aggregates and prion-like properties, are capable of activating the NLRP3–ASC inflammasome. In addition, we analyzed whether inhibition of ASC-dependent inflammasome activation is capable of inhibiting exogenously seeded and non-exogenously seeded Tau pathology in Tau transgenic mice in vivo.

## Materials and methods

### Animals

In this project, transgenic mice overexpressing the human (1N4R) Tau protein harboring the P301S mutation driven by the mouse Prion promoter (PS19, denoted TPS; The Jackson Laboratory, Bar Harbor, US) backcrossed to C57BL/6J background were used [87, 94, 101]. These mice exhibit a phenotype that mimics important aspects of Tauopathies. From the age of 11 months, filamentous Tau accumulates in the brain of these mice, and the mice subsequently develop a progressive neurodegenerative phenotype, characterized by clasping of the hind limbs, motoric problems, hippocampal atrophy, development of a hunchback and premature death [87, 101]. Tau seeding at the age of 3 months induces strong Tau-seeded Tau pathology already 7 weeks post-injection, absent in the parental strain at that age. In addition, mice deficient for ASC (backcrossed to C57BL/6J background) (Charles River Laboratory, Brussels, Belgium) were used to investigate the role of ASC inflammasome in Tau pathology [17, 69, 81]. Hemizygous TPS mice and hemizygous ASC knockout mice were crossed to obtain T +.ASC+/− , which were further crossed with ASC +/− mice to obtain T +.ASC−/− and T +.ASC +/+ littermates. Hemizygous ASC +/− mice were intercrossed to generate ASC −/− and ASC +/+ littermates for primary microglial cultures. All mice were genotyped by PCR analysis of tail biopsies. Animals were housed on a 12-h light/dark cycle in standard animal care facilities with access to food and water ad libitum. All animal experiments used in this study were performed in accordance with protocols approved by the institutional Ethics Committee for Animal Welfare.

### Primary microglial cultures

All cell culture reagents were purchased from Sigma-Aldrich (St. Louis, MO, USA) unless otherwise indicated. Primary microglial cultures were prepared from newborn (P0-2) mouse pups. The newborn mice were genotyped, euthanized by decapitation and dissected using standard procedures as described previously [17]. Briefly, brains were dissected, and meninges removed in ice-cold sterile HBSS medium under a Primo Vert light microscope (Zeiss, Oberkochen, Germany). The isolated cortices were transferred into chilled Dulbecco’s modified Eagle’s medium (DMEM), with 1% penicillin–streptomycin (PS; Invitrogen, Carlsbad, USA) followed by mechanical trituration, and subsequently filtered through a 70-μm cell strainer and centrifuged at 300 g for 5 min at 4 °C. The cell pellet was resuspended in DMEM supplemented with 10% horse serum, 10% fetal calf serum and 1% PS (DMEM 10:10:1) at 37 °C and seeded on poly-d-lysine-coated 175-cm^2^ culture flasks. After incubation in a humidified 5% CO_2_ at 37 °C for 10 days, the medium was replaced with fresh DMEM 10:10:1 supplemented with 1/3 colony stimulating factor 1 (CSF1) and incubated for 5 days at 37 °C and 5% CO_2_. At day 15, the microglial cells were removed by shaking the culture flasks for 3 h at 230 rpm at 37 °C in an orbital shaker. The supernatant was filtered through a 70-μm cell strainer and centrifuged for 10 min at 300 g and 4 °C. The cell pellet was resuspended in 1 ml DMEM 10:10:1 and counted with trypan blue. Cells were plated on poly-d-lysine-coated plates and coverslips and further used for experiments.

### Generation of “Tau seeds”

Tau seeds were generated as previously described [79, 87, 94]. Briefly, the human truncated 4R Tau, encompassing the four-repeat microtubule-binding domain with the P301L mutation and a Myc tag (K18-P301L; Q244-E372) was generated in *Escherichia coli*. Tau fragments (monomeric Tau; 67 µM) were incubated in a 1:2 ratio with low molecular weight heparin (MP Biomedicals, Santa Ana, CA, USA) in 100 mM ammonium acetate buffer (pH 7) at 37 °C for 5 days. The fibrillization mixture was centrifuged (100,000 g for 1 h at 4 °C) and the resultant pellet resuspended in the same buffer without heparin to a final concentration of 333 µM, aliquoted and stored at − 80 °C (“Tau seeds”). Successful Tau fibrillization was confirmed by Thioflavin T (Sigma-Aldrich, St. Louis, MO, USA) assay and immunoblotting. For all experiments, Tau seeds were sonicated (eight pulses of 30% amplitude) before use.

### Analysis of inflammatory activation in vitro

At the start of the experiment, fresh medium was added to the microglial culture. The cells were then primed with 1 μg/ml lipopolysaccharide (LPS) from *Escherichia coli* O26:B6 (Sigma-Aldrich) for 3 h at 37 °C and 5% CO_2_, washed with fresh medium and then treated with either 20 μM nigericin (Sigma-Aldrich) for 3 h or 5 μM of Tau seeds for 18 h, for each condition the medium was collected and the cells were fixed in 4% PFA in phosphate buffer saline (PBS). NLRP3 inhibitor MCC950 at 1 μM, or cathepsin B inhibitor CA-074 Me at 25 μM (both from Sigma-Aldrich), was added 15 min before treatment with either nigericin or Tau seeds. The various conditions were tested in both microglia cultures derived from ASC +/+ and ASC−/− mice minimally in three independent biological experiments.

### Mouse IL-1β ELISA

For measuring IL-1β concentrations, the Mouse IL-1β Ready-SET-GO! ELISA kit (eBioscience, San Diego, US) was used according to the manufacturer’s protocol. Briefly, a 96-well plate was coated overnight with anti-mouse IL-1β capture antibody, washed three times for 1 min each with PBS, 0.05% Tween 20, followed by blocking with 1 × ELISPOT diluent for 1 h at room temperature (RT), after which 100 μl of sample was applied per well. The standard curve [eight samples (from 1000 pg/ml to 8 μg/ml) provided in the kit] was included in duplicate in the analysis, as well as two blank controls. After incubation overnight at 4 °C, the anti-mouse IL-1β detection antibody was added for 1 h at RT, followed by incubation with avidin–HRP solution for 30 min at RT. Tetramethylbenzidine solution was used as a substrate and 1 M H_3_PO_4_ was used as a stop solution. The absorbances were read at 450 nm with a BioRad iMark microplate absorbance reader (BioRad, Hercules, US). The results were processed using GraphPad Prism software.

### Tau seeding experiments in vivo

To analyze the effect of Tau seeding on Tau pathology and the role of microglial inflammation, we performed injection of Tau seeds in TPS mice. The mice were deeply anesthetized by intraperitoneal injection of Ketamine/Xylazine mixture (Ketalar/Rompun) and placed in the stereotaxic apparatus (Kopf Instruments). Stereotactic injections of pre-aggregated Tau (Tau seeds) were performed as described previously [87]. Briefly, sonicated Tau seeds (5 µl; 333 µM) were injected using a 10-μl Hamilton syringe in the frontal cortex (A/P, + 2.0; L, + 1.4; D/V, − 1.0; relative to bregma) at a rate of 1 μl/min. After injection, the needle was kept in place for additional 5 min before gentle withdrawal. The injected mice were sacrificed at the indicated time post-injection for immunohistochemical analysis.

### Pharmacological inhibition by osmotic mini pump in Tau mice in vivo

To analyze the effect of the inflammasome inhibitor MCC950 (Sigma-Aldrich) on Tau-seeded Tau pathology, 3-month-old TPS mice were unilaterally injected into the right brain hemisphere with 5 µl (333 µM) Tau seeds (A/P, − 4.8; L, − 3.0; D/V, − 3.7; relative to bregma), as described above. In addition, an Alzet mini-osmotic pump (model 2006; Alzet, Cupertino, CA, US) attached via a catheter (20–25 mm) to a brain infusion cannula (brain infusion kit III; Alzet, Cupertino, CA, US) was implanted in a subcutaneous pocket towards the left hind limb as previously described [20]. The brain infusion cannula was slowly inserted through the skull into the right lateral ventricle (A/P, − 0.5; L, − 1.1; relative to bregma) and attached to the skull with two drops of adhesive (Loctite 454). The skin was sutured with a running horizontal mattress suture using 3-0 braided silk suture thread. The mice were monitored until complete recovery and were housed individually for 24 h. Thereafter the mice were monitored daily during the first week post-surgery and three times per week for 6 weeks. The filling of the mini-osmotic pumps and the preparation of the brain infusion assembly was performed according to the manufacturer’s instructions. The fully assembled mini-osmotic pumps were placed into a sterile 50-ml conical tube with 0.9% NaCl for priming at 37 °C for 60 h prior to implantation. The mini-osmotic pumps were filled with two different concentrations of MCC950 (to obtain a final concentration of 0.1 µM and of 0.5 µM) in sterile PBS. Calculations were based on a release rate of 0.15 µl/h from the mini-osmotic pumps into the total brain volume to obtain a final concentration of 0.1 µM and 0.5 µM of MCC950 in the brain (calculated for an estimated brain volume of 400 mm^**3**^). In the control mice, PBS was administered by mini-osmotic pump for 7 weeks. The treated mice were sacrificed 7 weeks post-injection.

### Immunohistochemical and immunocytological analysis

Immunohistological analysis was performed as described previously [79, 87, 94]. Briefly, the brains were dissected, after 2 min transcardiac perfusion with ice-cold PBS (Sigma-Aldrich, St. Louis, USA) and fixed for 24 h in 4% PFA–PBS at 4 °C. Free-floating sagittal sections (40 μm) were generated with a vibrating HM650 V microtome (Thermo Fisher Scientific, Waltham, MA, USA) and were preserved in PBS–sodium azide 0.1%. The sagittal brain sections were first washed twice in PBS and then three times in PBS + 0.1% Triton X-100 (PBST). Permeabilization of the tissue was performed using PBST + methanol (1:1) for 10 min, and subsequently blocked with PBST + 5% milk. Anti-Tau P-S202/T205 (AT8, 1:100; Thermo Fisher Scientific was used as a marker for Tau pathology and anti-Iba1 (Iba1, 1:500; Wako-Chemical GmbH, DE) was used as marker for microglial cells. The primary antibodies were incubated for 2 h at room temperature (RT) or overnight at 4 °C, followed by appropriate Alexa-coupled secondary antibody (1:500) in PBST + 5% milk for 1 h at RT. The slices were finally washed three times with PBST then twice with PBS and finally mounted with Fluoroprep mounting medium (BioMérieux, Marcy-l’Etoile, France). Staining with Thioflavin S (ThioS; Sigma-Aldrich), a specific β-sheet strand intercalant, and Gallyas silver (all chemicals from Sigma-Aldrich) staining were performed on vibratome sections as previously described [87] and were used to demonstrate the presence of NFTs in brain sections. For ThioS staining the brain slices were mounted on 3% gelatin-coated glass slides, washed twice in PBS and incubated for 5 min in 0.3% KMnO_4_. Subsequently, the slides were washed in a solution of 1% K_2_S_2_O_5_/1% oxalic acid until the brown color was removed, followed by a solution of 1% NaBH_4_ (prepared 2 h before use) for 20 s. Then the brain sections were incubated with 0.05% ThioS in 50% ethanol for 8 min, followed by two changes of 80% ethanol for 10 s each and three washes with large volumes of demineralized water. Slides were then placed in a high-concentrated phosphate buffer overnight in dark at 4 °C. For silver staining the free-floating brain sections were washed in demineralized water and placed for 5 min in 5% periodic acid solution, then washed twice in demineralized water and treated for 1 min with an alkaline silver iodide solution (1 M NaOH, 0.6 M KI, 0.035% silver nitrate). Subsequently, the brain slices were washed twice for 5 min with 0.5% acetic acid solution and placed in developer solution (combining solutions A—0.5% sodium carbonate:B—0.025 M NH_4_NO_3_, 0.012 M AgNO_3_, 0.0035 M tungstosilicic acid:C—0.025 M NH_4_NO_3_, 0.012 M AgNO_3_, 0.0035 M tungstosilicic acid, 0.28% formaldehyde in a 10:3:7 ratio) for 5 min. Then the brain slices were rinsed twice in 0.5% acetic acid, washed with demineralized water and placed in 0.1% gold chloride solution for 5 min followed by a 1% sodium thiosulphate solution for 5 min and a final wash in water. All chemicals used were from Sigma-Aldrich. Immunocytochemistry on primary microglial cells was performed similarly as above, using the lysosomal marker anti-LAMP-1 antibody (clone H4A3, 1:50; Santa Cruz Biotechnology, Dallas, TX, USA) and anti-c-Myc antibody (1:50; Sigma-Aldrich) for Tau seeds. Fixation of the cells was performed with 4% PFA in PBS for 10 min at RT. Image acquisition was performed with a Leica DM450B fluorescence microscope (Leica, Wetzlar, DE), EVOS FL Auto Imaging System (Thermo Fisher Scientific) and standard light microscope. Image analysis was performed with Image J (National Institutes of Health) blinded to the genotype and/or treatment of the mice. Briefly, the fluorescent TIFF images were converted to 16-bit images and then thresholded using the Image J Default method to allow quantification of the stained area without detection of the background staining; the threshold was then applied to all sections. For quantification of the silver staining area, the 16-bit images were inverted prior to the same thresholding procedure as above. The amount of staining was measured as the percentage of area occupied by positive signal within the brain region (frontal cortex, hippocampal CA1). The results were statistically processed with GraphPad Prism 7.04 software (GraphPad, San Diego, CA, USA).

### Statistical analysis

The number of samples or animals is specified in the caption for each experiment. Results are expressed as the mean ± SEM. Statistical analysis was performed using Mann–Whitney *t* test and one-way ANOVA or two-way ANOVA with Dunnett’s/Tukey’s multiple comparison post hoc test (specified in the accompanying figure legend). All analyses were performed using GraphPad Prism software (GraphPad Software Inc). Statistical significance was defined as *P* < 0.05.

## Results

### Aggregated Tau seeds activate NLRP3–ASC inflammasome in primary microglia

**Fig. 1:**
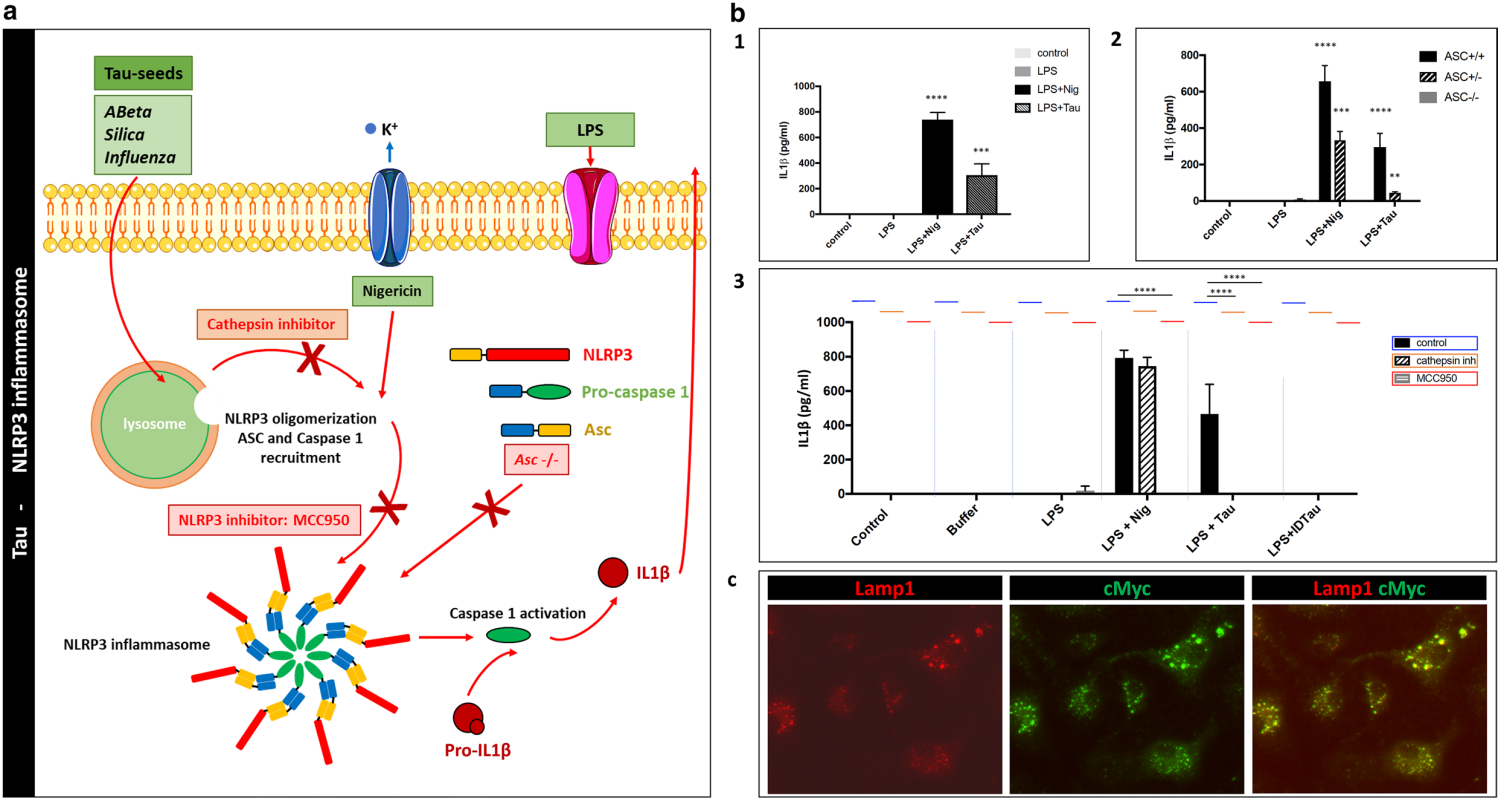
Pre-aggregated Tau seeds activate NLRP3–ASC inflammasome microglia following endo-lysosomal sorting. **a** Schematic presentation of the NLRP3–ASC inflammasome pathway indicating the experiments used to interfere with this pathway to study the pathway of NLRP3–ASC inflammasome activation by Tau seeds (some of the graphical elements were adapted from Servier Medical Art—https://smart.servier.com/). **b** (1) Measurements of IL-1β concentrations in the medium of primary microglia following the addition of buffer (control), LPS, LPS + nigericin (LPS + Nig) and LPS + Tau seeds (LPS + Tau) demonstrate strong induction of IL-1β secretion by the latter two conditions (three independent experiments, *n* = 3 per experiment and per condition; one-way ANOVA with Dunnett’s multiple comparison post hoc test compared to control, ***p value < 0.001, ****p < 0.0001). **b** (2) Measurements of IL-1β concentrations in the medium of primary microglial cultures following addition of buffer (control), LPS, LPS + nigericin (LPS + Nig) and LPS + Tau seeds (LPS + Tau); ASC +/− and ASC +/+ primary microglial cultures demonstrates gene-dosage dependent induction of IL-1β, while induction in ASC−/− microglia is strongly inhibited (three independent experiments, *n* = 3 per experiment and per condition; two-way ANOVA with Dunnett’s multiple comparison post hoc test; LPS + Nig: ASC +/+ vs ASC−/−, ****p < 0.0001; ASC +/+ vs ASC +/– , ***p value < 0.001; LPS + Tau: ASC +/+ vs ASC−/−, ****p < 0.0001; ASC +/+ vs ASC +/– , **p value < 0.01). **b** (3) Measurements of IL-1β concentrations in the medium of primary microglia in control conditions (control) and following addition of buffer only (buffer), LPS, LPS + nigericin (LPS + Nig) and LPS + Tau seeds (LPS + Tau), in the absence (blue lines) or presence of MCC950 (red lines), a pharmacological inhibitor of NLRP3 inflammasome, demonstrate strong induction of IL-1β secretion by the latter two conditions in the absence of MCC950, while induction of IL-1β secretion in the presence of MCC950 is strongly inhibited. This indicates the dependence on NLRP3 activation. Measurements of IL-1β concentrations in the presence and absence of Cathepsin B inhibitor (CA-074 Me) (orange lines) demonstrates strong induction of IL-1β secretion in the medium of primary microglia following addition of LPS + nigericin (LPS + Nig) and LPS + Tau seeds (LPS + Tau) in the absence of the cathepsin B inhibitor, while induction of IL-1β secretion in the presence of cathepsin inhibitor is strongly inhibited for LPS + Tau seeds. This indicates the dependence of NLRP3–ASC inflammasome activation by Tau seeds on cathepsin B activity (three independent biological experiments; *n* = 3 or 2 samples per experiment and per condition; two-way ANOVA with Tukey’s multiple comparison post hoc test; ****p < 0.0001). **c** Immunocytological analysis reveals strong co-localization of Tau seeds and microglial cells, respectively, stained with anti-myc antibody (green) and with anti-LAMP1 antibody (red), a lysosomal marker. This demonstrates uptake of Tau seeds and sorting to lysosomal compartments

The inflammasomes are innate immune system sensors that regulate microglial activation in response to danger signals [35]. In this process, the NLRP3–ASC inflammasome becomes activated by a dual stimuli leading to heteromer formation of ASC and activation of caspase-1 [35]. Subsequently, activated caspase-1 cleaves pro-IL-1β to IL-1β, which is secreted and induces a pro-inflammatory reaction and microglial activation (Fig. 1a). We here analyzed whether pre-aggregated Tau could activate microglia in an ASC-dependent way. Hereto, we used pre-aggregated Tau fragments, encompassing the microtubule-binding domain (MTBD) of Tau, previously demonstrated to efficiently seed Tau aggregation in our well-characterized in vitro and in vivo models [79, 87, 94, 98], further referred to as Tau seeds. We added Tau seeds to LPS-primed primary microglia, and measured IL-1β secretion as the result of inflammasome activation. As inflammasome activation requires a dual stimuli, we used LPS priming. Addition of nigericin after LPS priming (LPS + Nig) was used as known activator of ASC inflammasome in microglia. Measurement of the IL-1β concentrations demonstrated a clear-cut induction of IL-1β secretion not only following stimulation with LPS and nigericin, but also following application of Tau seeds after LPS priming. In contrast, buffer or LPS treatment alone did not result in IL-1β release (Fig. 1b1). To assess whether induction of IL-1β secretion by Tau seeds was ASC dependent, we combined LPS treatment with Tau seeds or nigericin in primary microglia derived from wild-type and ASC-deficient mice, respectively. Addition of Tau seeds or nigericin to LPS-primed ASC-deficient microglia did not induce IL-1β secretion in contrast to wild-type microglia (Fig. 1b2), indicating ASC dependency.

To further assess the upstream pathway of Tau-seed dependent ASC inflammasome activation, we used MCC950, a well-characterized inhibitor of the NLRP3 inflammasome [16]. Addition of MCC950 inhibited induction of IL-1β secretion following the addition of Tau seeds to LPS-primed microglia (Fig. 1b3), demonstrating activation of NLRP3–ASC inflammasome by Tau seeds. We further identified the upstream pathway of inflammasome activation by Tau seeds in microglia, using immunocytological analysis, demonstrating strong co-localization of Tau seeds with LAMP1, a well-accepted lysosomal marker (Fig. 1c). We thereby demonstrate that Tau seeds are efficiently taken up by primary microglia and sorted to lysosomes. In view of the well-characterized involvement of cathepsin B release from lysosomes in NLRP3 activation, we further assessed the involvement of cathepsin B release in the activation of the inflammasome by aggregated Tau. Hereto, the specific cathepsin B inhibitor (CA-074-Me) was used, which inhibited IL-1β secretion induced by Tau seeds (Fig. 1b3). Taken together, our data indicate that Tau seeds activate NLRP3–ASC inflammasome following microglial uptake, lysosomal destabilization and Cathepsin B release.

Our data demonstrate that pre-aggregated Tau activates NLRP3–ASC inflammasome similar as previously demonstrated for aggregated Aβ, through a similar pathway involving lysosomal perturbation. Interestingly, inhibition of inflammasome activation was shown to decrease amyloid pathology in vivo. We, therefore, further assessed the relevance of our findings on modulation of Tau pathology in vivo and analyzed whether inflammasome deficiency affects Tau-seed induced Tau pathology in TauP301S transgenic mice.

### ASC inflammasome modulates exogenously seeded Tau pathology in Tau transgenic mice

To investigate the role of ASC inflammasome activation on exogenously seeded Tau pathology in vivo, we used our previously developed and well-characterized Tau-seeding models [79, 87, 94]. To model prion-like or templated Tau-seeded Tau pathology, TauP301S (PS19, denoted TPS) transgenic mice were stereotactically injected in frontal cortex with Tau seeds. Tau seeding using intracerebral injection of pre-aggregated Tau seeds at 3 months of age induces strong Tau pathology in Tau transgenic mice, dramatically accelerating and exacerbating Tau pathology in this model. Abundant mature neurofibrillary tangles are detected in the cortex of Tau-seeded Tau transgenic mice following Tau seeding, while absent in age-matched non-seeded Tau transgenic mice as shown in suppl. Figure 1 (Online Resource 1a), confirming our previously published data [79, 87, 94]. Injection of non-aggregated (monomeric) Tau fragments (Online Resource 1b) does not induce significant Tau-seeded Tau aggregation, highlighting the importance of pre-aggregation of Tau for inducing Tau seeding. Importantly, in the absence of Tau seeding, Tau pathology is only detected from 11 months onwards in this model.

**Fig. 2:**
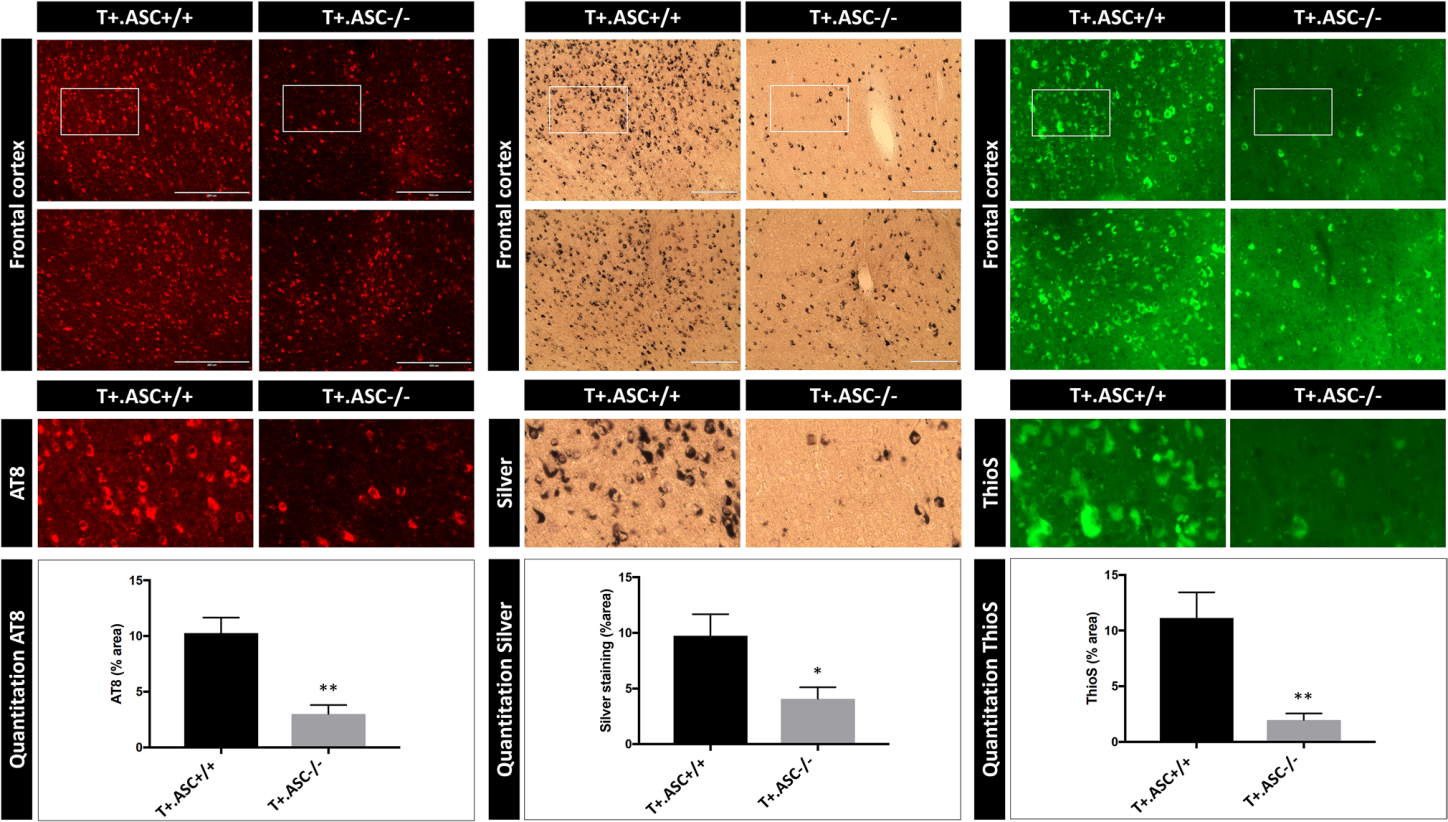
ASC deficiency reduces induction of Tau pathology following exogenous Tau seeding in Tau transgenic mice. Immunohistological analysis of Tau pathology using anti-P-Tau (AT8) antibody in brains of T +. ASC +/+ and T +.ASC−/− mice. Tau seeding was performed at 3 months of age and analyzed 6 months post-seeding. Representative images of AT8 staining (red) in frontal cortex in T +.ASC +/+ and T +.ASC−/− mice are presented. Quantitative analysis of the area stained with AT8 reveals a significantly lower induction of Tau pathology in T +.ASC−/− compared to T +.ASC +/+ mice (*n* = 7, T +.ASC +/+; *n* = 5, T +.ASC−/−; Mann–Whitney *t* test; **p < 0.01). Silver staining (black) and ThioS (green) staining demonstrate the presence of mature NFTs following Tau seeding in Tau transgenic mice, representative images of frontal cortex are shown. Tau-seeding induced Tau pathology (NFTs) assessed by silver and ThioS staining was significantly less in T +.ASC−/− compared to T +.ASC +/+ mice (*n* = 7, T +.ASC +/+; *n* = 5, T +.ASC−/−; Mann–Whitney *t* test; *p < 0.05, **p < 0.01). These data confirmed an independent pilot study performed prior to this study to explore the potential effect of ASC deficiency on Tau-seeded Tau pathology, using Tau seeding in T +.ASC−/− compared to T +.ASC +/+ mice, demonstrating significantly inhibited Tau pathology following Tau seeding in ASC-deficient Tau mice (Online Resource 2)

To investigate the modulatory role of ASC on induction of Tau pathology by Tau seeding, we performed Tau seeding in Tau transgenic mice deficient for ASC, as crucial component of the inflammasome [17, 35, 69]. Tau transgenic mice were crossed with ASC-deficient mice to generate, respectively ASC-deficient Tau mice (T +.ASC−/−) and ASC-expressing Tau mice (T +.ASC +/+). T +.ASC +/+ and T +.ASC−/− mice were stereotactically injected in frontal cortex with Tau seeds. Immunohistochemical analysis of Tau-seeding induced Tau aggregation was performed using our previously optimized AT8 staining protocol strongly correlating with aggregated Tau [87]. This revealed a decreased Tau-seed induced Tau pathology in ASC-deficient Tau transgenic mice (T +.ASC−/−) compared to ASC-expressing Tau transgenic mice (T +.ASC +/+) (Fig. 2). Quantitative analysis measuring the area of AT8 staining demonstrated significantly reduced induction of Tau pathology by Tau seeding in brains of T +.ASC−/− mice compared to T +.ASC +/+ mice (Fig. 2). These findings were further confirmed by Gallyas silver staining and Thioflavin S (ThioS) staining to stain mature NFTs, revealing decreased mature NFTs in Tau-seeded T +.ASC−/− mice compared to T +.ASC +/+ mice (Fig. 2). Decreased induction of Tau-seeded Tau pathology was also detected at the contralateral side in T + ASC−/− compared to T +.ASC +/+ mice, data presented in suppl. Figure 2 (Online Resource 2b), as Tau pathology spreads in this model to the contralateral side. Importantly, our analysis concerning the effect of ASC on Tau-seeded Tau pathology was performed based on a previously performed pilot study in a different cohort of T +.ASC +/+ and T +.ASC−/− mice exposed to Tau seeding. In both independent analyses, we found significantly inhibited exogenously seeded Tau pathology in T +.ASC−/− mice compared to T +.ASC +/+ mice, highlighting the consistency of the observed results (Online Resource 2).

Hence, we here demonstrate in T +.ASC−/− and T +.ASC +/+ mice that ASC, a crucial component of the inflammasome, contributes to exogenously Tau-seeded Tau pathology and that ASC deficiency inhibits exogenously seeded Tau pathology.

### Inhibition of NLRP3, upstream activator of ASC inflammasome, inhibits exogenously seeded Tau pathology

**Fig. 3:**
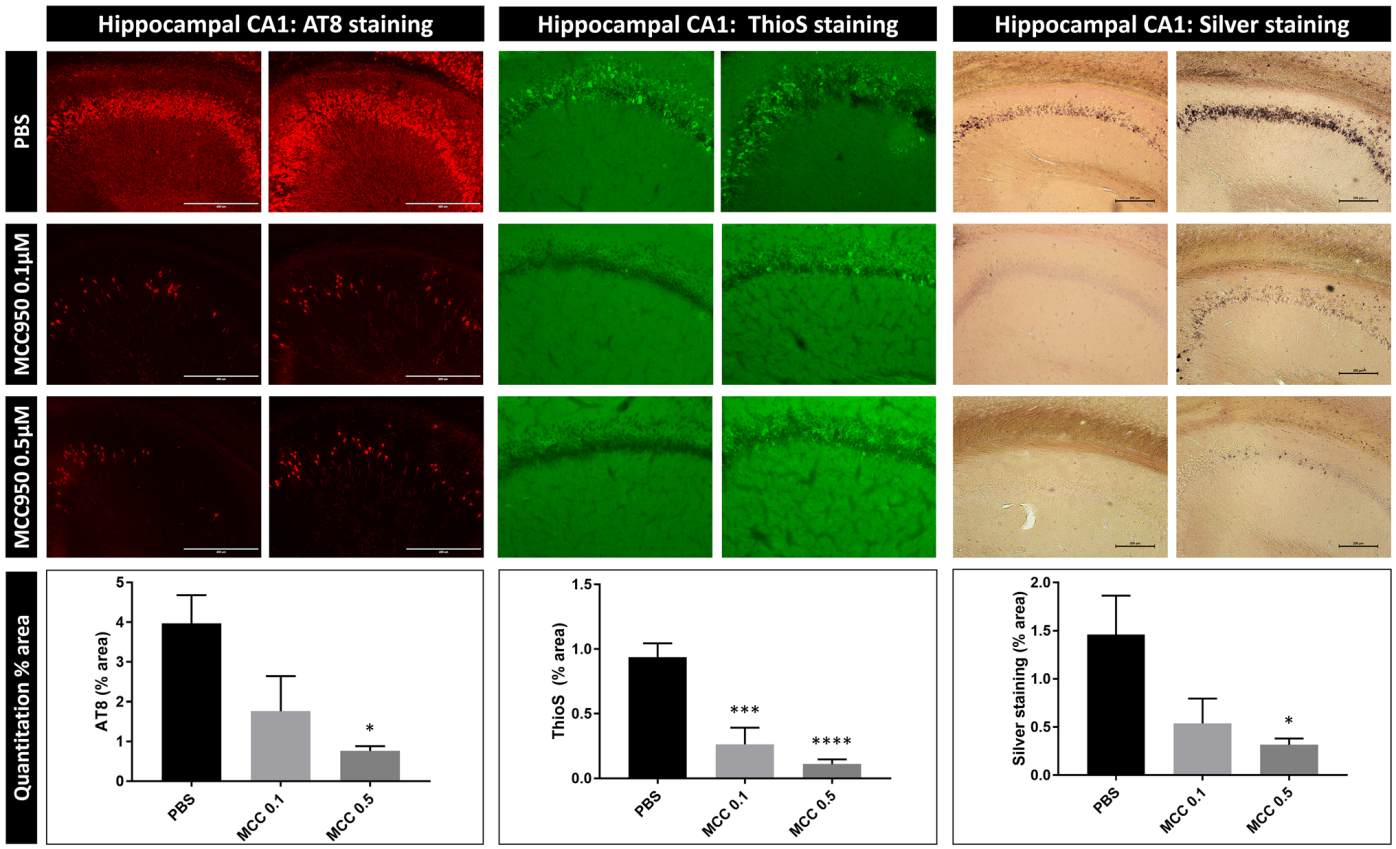
Pharmacological inhibition of the inflammasome reduces Tau pathology induced by Tau seeding in Tau transgenic mice, demonstrating therapeutic potential and NLRP3 inflammasome involvement. Immunohistological analysis of Tau pathology in brains of Tau transgenic mice 7 weeks post-Tau seeding. Tau seeding was performed at 3 months of age and analyzed 7 weeks post-seeding. Tau mice were chronically treated with a final concentration of 0.1 µM MCC950, 0.5 µM MCC950 and with PBS using osmotic mini-pumps for 7 weeks. Representative images of anti-P-Tau (AT8; red) staining in frontal cortex in chronically treated mice with 0.1 µM MCC950, with 0.5 µM MCC950 and with PBS, reveal significantly reduced Tau pathology in 0.5 µM MCC950-treated mice compared to PBS-treated mice. Quantitative analysis of the area stained with AT8 reveals a significantly lower induction of Tau pathology in 0.5 µM MCC950-treated mice compared to PBS-treated mice (*n* = 8, PBS; *n* = 8, 0.1 µM MCC950; *n* = 6, 0.5 µM MCC950; one-way ANOVA Dunnett’s multiple comparison; 0.1 µM MCC950 vs. PBS, *p* = 0.0630; 0.5 µM MCC950 vs. PBS, *p < 0.05). Silver (black) and ThioS (green) staining demonstrate the presence of mature NFTs following Tau seeding in Tau transgenic mice; representative images of frontal cortex are shown. Silver and ThioS staining showed significantly less Tau-seeding induced Tau pathology following 0.5 µM MCC950 treatment compared to PBS (*n* = 8, PBS; *n* = 8, 0.1 µM MCC950; *n* = 6, 0.5 µM MCC950; one-way ANOVA Dunnett’s multiple comparison; ThioS: 0.5 µM MCC950 vs. PBS, ****p < 0.0001; ThioS: 0.1 µM MCC950 vs. PBS, ***p < 0.001; silver: 0.5 µM MCC950 vs. PBS, *p < 0.05; silver: 0.1 µM MCC950 vs. PBS *p* = 0.0681)

Our findings indicate a modulatory role of the ASC inflammasome in exogenously seeded Tau pathology and suggest a therapeutic potential of pharmacological inflammasome inhibition to target seeding of Tau pathology. Our in vitro analysis delineated microglial uptake of Tau seeds, lysosomal sorting and NLRP3 activation as upstream pathway for ASC inflammasome activation. We, therefore, assessed the implication of the NLRP3 inflammasome in the modulatory role of ASC on Tau pathology in vivo. To elaborate the therapeutic potential of our findings, we used a previously characterized pharmacological inhibitor of the NLRP3 inflammasome MCC950, known to block NLRP3–ASC-induced IL-1β secretion and ASC oligomerization [16]. Tau transgenic mice were stereotactically injected with Tau seeds and were chronically treated with the inflammasome inhibitor MCC950, using two different doses (0.5 μM MCC950 and 0.1 μM MCC950, respectively). The inflammasome inhibitor MCC950 was chronically administered intracerebrally using osmotic mini-pumps for 7 weeks and chronic administration of vehicle with osmotic pumps was used as control. Tau-seeded Tau pathology was analyzed 7 weeks post-injection as this is the maximal time span for the mini-osmotic pumps without replacement. AT8 immunostaining revealed strongly decreased induction of Tau pathology following exogenous seeding upon chronic administration of MCC950 compared to control PBS treatment (Fig. 3). Quantification of the results demonstrated significantly reduced Tau pathology in mice treated with 0.5 μM MCC950 compared to the control mice (Fig. 3). Mice chronically treated with 0.1 μM MCC950 displayed a strong tendency towards decreased Tau pathology but not reaching significance. Taken together, a dose-dependent decrease in Tau-seeded Tau pathology was demonstrated following chronic pharmacological NLRP3 inhibition.

### Exogenously seeded Tau pathology in Tau transgenic mice is associated with microgliosis, which is decreased in ASC-deficient Tau mice

**Fig. 4:**
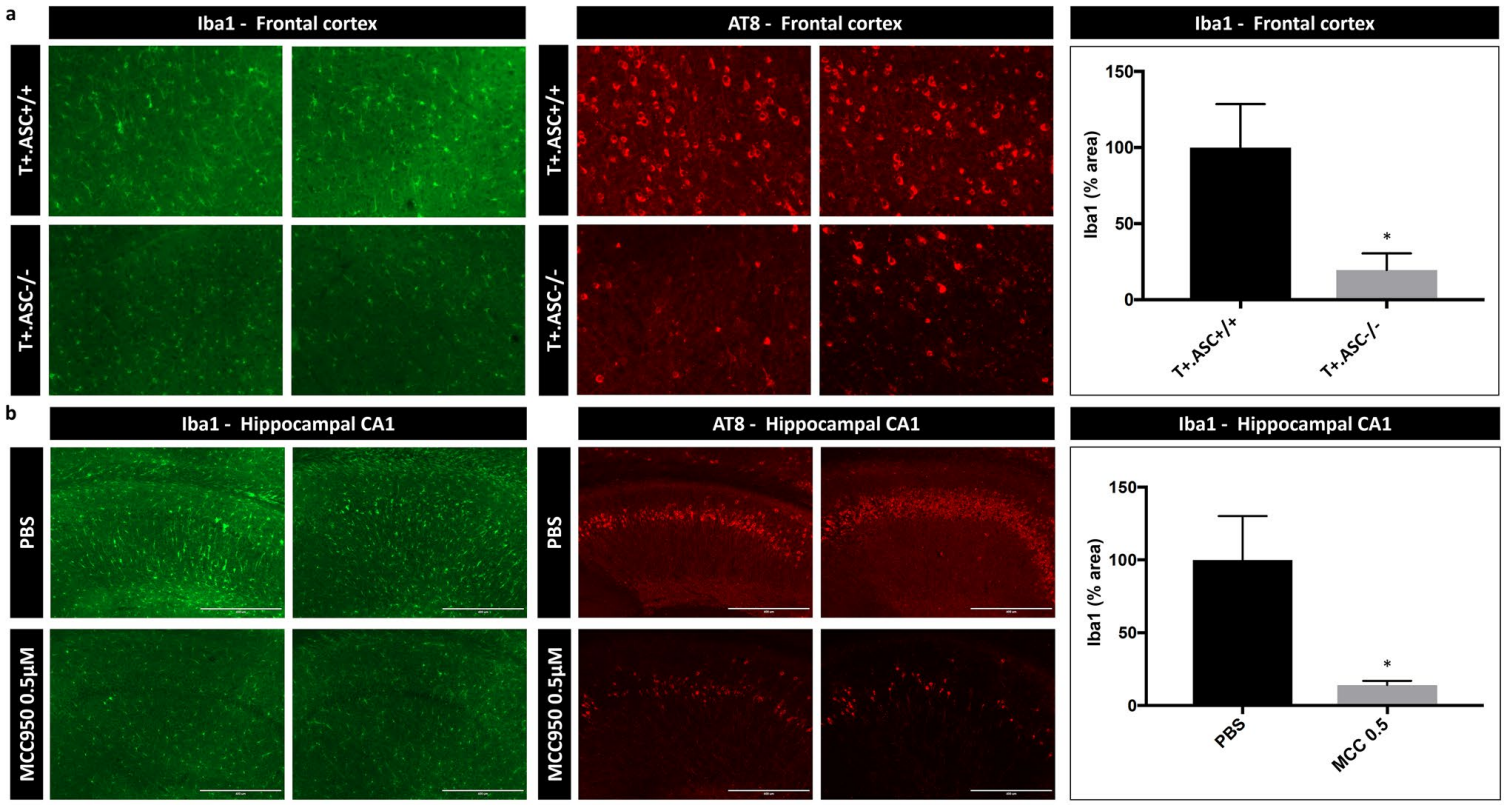
Tau-seeding induced microgliosis is decreased in ASC-deficient Tau mice. **a** Representative images of immunohistological staining using Iba1 (green) and anti-P-Tau (AT8; red) following Tau seeding in frontal cortex of T +.ASC-/- and T +. ASC +/+ mice are presented. Quantitative analysis of anti-Iba1 staining demonstrates lower microglial activation in T +. ASC−/− compared to T +.ASC +/+ mice (*n* = 7 T +.ASC +/+ and *n* = 5 T +.ASC−/− mice; Mann–Whitney *t* test; *p < 0.05). **b** Representative images of immunohistological staining using Iba1 (green) and AT8 (red) following Tau seeding in mice chronically treated with PBS or with 0.5 µM MCC950 for 7 weeks are presented. Quantitative analysis of anti-Iba1 staining demonstrates lower microglial activation upon exogenous Tau seeding in the presence of chronic 0.5 µM MCC950 treatment compared to PBS treatment in Tau transgenic mice (*n* = 8, PBS treated; *n* = 6, 0.5 µM MCC950 treated; Mann–Whitney *t* test; *p < 0.05)

We next analyzed the effect of Tau-seeding induced Tau pathology on microglial activation in Tau mice in vivo and its dependency on ASC inflammasome. We performed combined immunohistological analysis with anti-phospho-Tau antibody AT8 and anti-Iba1 antibody in brains of Tau mice with Tau-seed induced Tau pathology. We found that exogenously seeded Tau pathology is associated with microglial activation, reflected in increased Iba1 staining intensity and the presence of microglial cells with altered morphology indicative of microglial activation (Fig. 4a and suppl. Figure 3—Online Resource 3). Interestingly, microglial activation assessed by Iba1 staining following Tau seeding in T +.ASC−/− mice was significantly decreased compared to T +.ASC +/+ mice (Fig. 4a). We also analyzed microglial activation using Iba1 staining in exogenously seeded Tau mice following chronic administration of MCC950. This revealed significantly reduced Iba1 staining following chronic administration of 0.5 µM MCC950 using mini- osmotic pumps (Fig. 4b). Taken together, ASC deficiency and pharmacological NLRP3 inhibition significantly decreased microglial activation associated with exogenously seeded Tau pathology in Tau mice in vivo. It must be noted that within the current paradigm we cannot determine whether microgliosis is decreased due to the fact that less Tau pathology is present or to inhibition of microglial activation by NLRP3–ASC-dependent inflammasome inhibition or deficiency, or a combination of both.

### ASC inflammasome modulates non-exogenously seeded Tau pathology in Tau transgenic mice in vivo

**Fig. 5:**
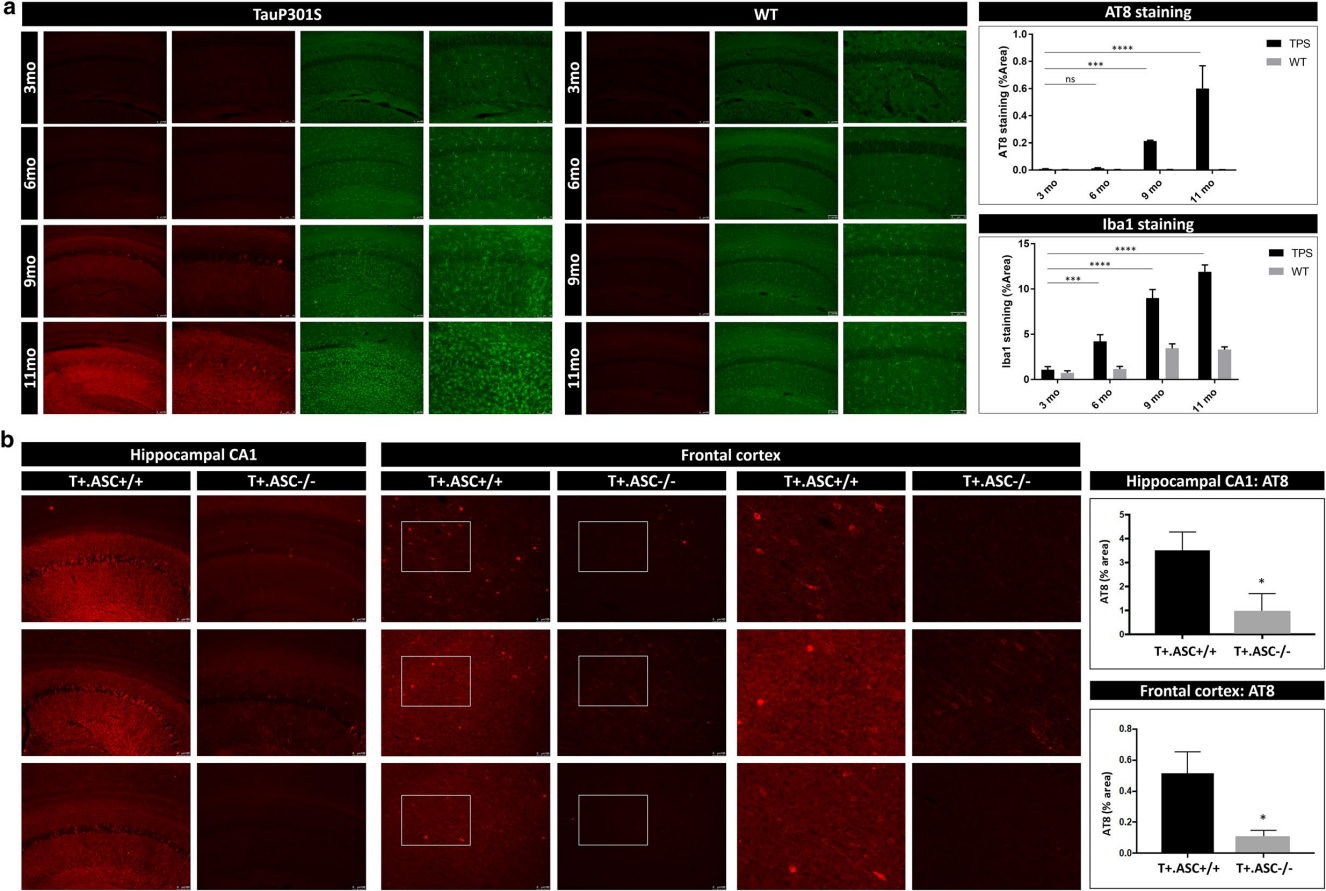
Age-dependent increase in microglial activation in Tau transgenic mice and decreased Tau pathology in ASC-deficient Tau mice indicate a contribution of the inflammasome in non-exogenously seeded Tau pathology. **a** Representative images of Iba1 (green) and anti-P-Tau (AT8; red) staining in hippocampal CA1 region of Tau mice (TauP301S/TPS) and wild-type (WT) littermates of 3 months, 6 months, 9 months and 11 months. Quantitative analysis reveals increased microgliosis in Tau transgenic mice, preceding overt mature neurofibrillary tangles. Although the mature NFTs are detected scarcely from 9 months onwards and are more frequently from 11 to 12 months onwards (AT8 area; *n* = 6, TPS and WT of 3 and 6 months; *n* = 4, TPS of 9 months; *n* = 5, WT of 9 months; *n* = 3, TPS and WT of 11 months; two-way ANOVA with Dunnett’s multiple comparison post hoc test, ***p < 0,001, ****p < 0.0001), increased microglial activation is already detected from 6 months onwards (Iba1 area; *n* = 6, TPS and WT of 3 and 6 months; *n* = 4, TPS of 9 months; *n* = 5, WT of 9 months; *n* = 3, TPS and WT of 11 months; two-way ANOVA with Dunnett’s multiple comparison post hoc test, ***p < 0,001, ****p < 0,0001). **b** Representative images of Tau pathology assessed using anti-P-Tau (AT8; red) staining in hippocampal CA1 and frontal cortical regions of 11-month-old T +. ASC +/+ and T +.ASC−/− mice are presented. Quantitative analysis revealed decreased Tau pathology in non-exogenously seeded T +.ASC−/− mice compared to T +. ASC +/+ mice (*n* = 6, T +.ASC +/+; *n* = 6, T +.ASC−/−; Mann–Whitney *t* test, *p < 0.05)

Using a sensitive Tau seed reporter assay, endogenous Tau seeds have been identified in brains of Tau transgenic mice and have been shown to precede Tau aggregation [45, 53, 84]. TauP301S transgenic mice develop a neurodegenerative phenotype starting at the age of around 11 months [88, 101]. In the absence of Tau seeding, scarce Tau pathology starts to develop at the age of 11 months, starting with scarce NFTs in hippocampus and cortex, progressively worsening and followed by progressive clasping and motor symptoms. Interestingly, microgliosis was previously demonstrated to precede mature NFTs and to exacerbate Tau pathology in the TauP301S model used in this work [101]. We, therefore, analyzed whether ASC deficiency could modulate non-exogenously seeded Tau pathology in this model. We first analyzed the spatio-temporal progression of microglial activation in relation to Tau pathology development in TauP301S mice, using Iba1 staining in different age groups. Microglial activation strongly correlated with but preceded overt and mature Tau neurofibrillary tangles (Fig. 5a). Whether this microglial activation is induced by an excreted factor of Tau—e.g., Tau seeds, released or extracellularly presented Tau fragments—or by an alternative factor different from Tau is currently unclear. Our data indicate that microgliosis precedes overt mature NFTs in this TauP301S model, corroborating previous findings. In addition, co-staining using anti-Iba1 and AT8 revealed the presence of AT8-positive puncta in activated microglia in Tau transgenic mice, as shown in suppl. Figure 4 (Online Resource 4b, d). Using immunohistological staining, we furthermore demonstrated that microgliosis in Tau transgenic mice was associated with a punctate staining of ASC and NLRP3, indicative of complex formation and inflammasome activation not detected in non-transgenic mice (Online Resource 4a, c). Next, we assessed whether modulation of microglial activation in Tau transgenic mice by ASC inflammasome deficiency affected the progression of Tau pathology in the absence of exogenous Tau seeding, by comparative analysis of Tau pathology in brains of T +.ASC +/+ mice and T +.ASC−/− mice at the age of 11 months using AT8 staining. This analysis revealed a significant decrease in Tau pathology in T +.ASC−/− mice compared to T +.ASC +/+ mice (Fig. 5b). Hence, our data demonstrate that ASC inflammasome exacerbates non-exogenously seeded Tau pathology in Tau transgenic mice in vivo.

## Discussion

**Fig. 6:**
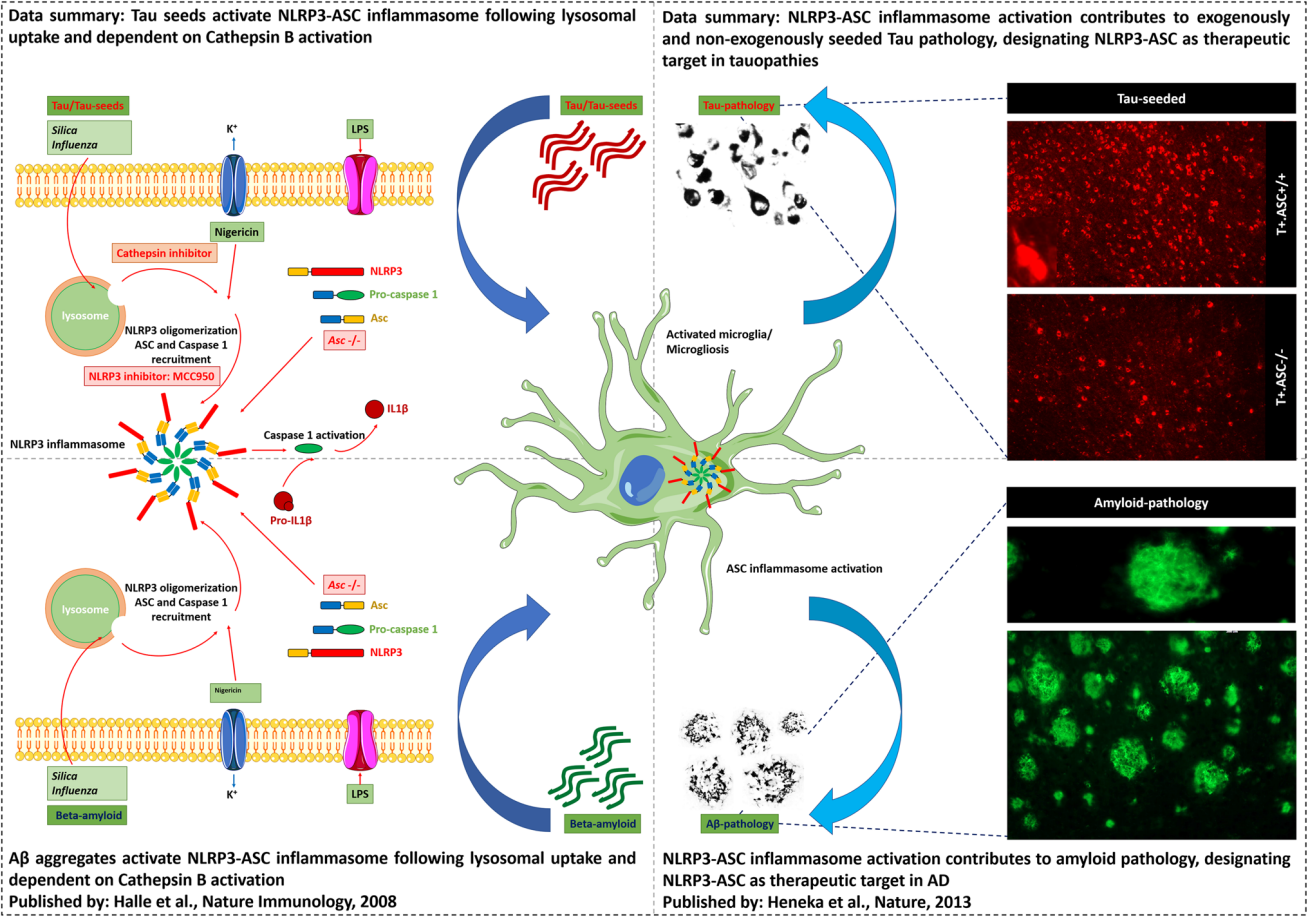
Data summary. Schematic summary of the data in relation with previously published data. Tau seeds, like aggregated Aβ (Halle et al.), activate NLRP3–ASC inflammasome following lysosomal uptake and dependent on Cathepsin B activation. NLRP3–ASC inflammasome activation contributes to exogenously and non-exogenously seeded Tau pathology, designating NLRP3–ASC as therapeutic target in Tauopathies. Previously, Heneka et al. showed that NLRP3–ASC inflammasome activation exacerbates amyloid pathology in vivo

In this work, we analyzed the link between inflammasome and Tau pathology (Fig. 6), as Tau pathology is invariably associated with microglial alterations in patients and in in vivo models. We here (i) identified aggregated Tau acting as prion-like seeds as a novel activator of NLRP3–ASC inflammasome. We furthermore (ii) demonstrate that NLRP3–ASC inflammasome activation exacerbates exogenously seeded Tau pathology and (iii) non-exogenously seeded Tau pathology in vivo. Our findings thereby provide novel insights into the contributing mechanisms of Tau-induced microgliosis and its contribution to prion-like seeding of Tau pathology. Our findings further support a role for microglia not as mere bystanders in Tau-seeded Tau pathology but as active modulators of the pathogenetic process and pinpoint a novel target for Tau-seeded Tau pathology. The NLRP3–ASC inflammasome (iv), scrutinized as therapeutic target for many disorders and previously identified as Aβ-related therapeutic target, is herewith identified as a target for Tauopathies including AD. Importantly, the NLRP3–ASC inflammasome pathway thereby presents as a highly interesting target, concomitantly positively modulating three key pathogenetic features of AD, i.e., Tau pathology, amyloid pathology and neuroinflammation.

### Aggregated Tau activates the NLRP3–ASC inflammasome

Here we identified aggregated Tau seeds as a novel activator of the NLRP3–ASC inflammasome. The NLRP3 inflammasome can be activated by structurally diverse stimuli including ATP, imidazoquinoline derivatives, various crystals as well as by bacterial toxins [21, 51, 70, 72]. Elegant work demonstrated that aggregated Aβ displaying amyloid structure [38] activates the NLRP3–ASC inflammasome. This finding is particularly interesting in view of the existence of a panoply of amyloids ranging from bacterial amyloids to amyloids with physiological functions as well as pathogenetic roles [22, 34, 54, 55]. Halle et al. suggested that different aggregated proteins displaying amyloid structure involved in protein aggregation-associated disorders, therefore, could be considered as potential inflammasome activator [38, 41, 42]. This hypothesis was further corroborated by the identification of islet amyloid polypeptide (iAPP) as NLRP3 inflammasome activator, as well as aggregated alpha-synuclein [15, 73]. Importantly, our current data indicating aggregated Tau as activator of NLRP3–ASC inflammasome further lend support to this hypothesis extending a potential role of NLRP3–ASC inflammasome beyond AD and primary Tauopathies to a variety of neurodegenerative disorders characterized by aggregating proteins [41]. While the role of inflammasome activation in neurological disorders is increasingly emerging, we here demonstrate that aggregated Tau activates the NLRP3–ASC inflammasome, providing a compelling molecular mechanism for the close and invariable association of microglial changes and Tau pathology in Tauopathies.

### Tau activates NLRP3–ASC inflammasome: a compelling molecular mechanism for Tau-associated neuroinflammatory changes in AD and Tauopathies

Tau aggregation is closely associated with microglial changes in AD and Tauopathies [5, 46, 64, 80, 99]. While neuroinflammatory changes are prominent in AD, they are not restricted to AD, and are also present in primary Tauopathies, in the absence of amyloid plaques [5, 46, 64, 80, 99]. Microglial alterations have furthermore been demonstrated in different Tauopathies using postmortem analysis [5, 46, 64, 80, 99] and using in vivo PET analysis to strongly correlate with Tau pathology in Tauopathies including CBD, PSP and FTD [27, 28, 67]. Also, in AD, PET imaging demonstrated strong correlation between microgliosis and NFTs and NT in brains of AD patients [67]. Neuroinflammatory changes associated with Tau pathology include not only altered chemokine and cytokine profiles and microglial activation, but also microglial degeneration [5, 18, 46, 64, 86, 90, 100]. While detailed analysis of Tau-associated neuroinflammatory processes will yield insight into their association with different stages of the disease process, these changes are in line with a potential role for inflammasome activation by Tau. We here identified a molecular mechanism, which contributes to the observed association between microglial changes and Tau pathology animal models, by the identification of NLRP3–ASC inflammasome activation as a contributor to microglial activation associated with Tau pathology. Tau-induced NLRP3–ASC inflammasome activation provides a compelling mechanism for the association between microglial activation and Tau pathology observed in AD and related Tauopathies.

### Aggregated Tau activates the inflammasome by a similar pathway as Aβ, implicating microglial uptake, lysosomal sorting and the NLRP3–ASC axis

Aggregated Aβ was recently demonstrated to activate NLRP3–ASC inflammasome [38], requiring microglial uptake of aggregated Aβ, subsequent lysosomal sorting and rupture, followed by Cathepsin B release activating NLRP3–ASC inflammasome [38]. Here we identified Tau-seed induced activation of the NLRP3–ASC inflammasome. NLRP3–ASC inflammasome activation in general occurs as a response to danger signals, resulting in the activation of NLRP3 receptor, formation of ASC, inducing the cleavage of pro-caspase-1 into active caspase-1, resulting in cleavage of pro-IL-1β into IL-1β which is subsequently secreted and resulting in microglial activation [35, 40, 41, 59, 71]. We have demonstrated that Tau seeds induced IL-1β secretion in an ASC-dependent way, as the process was inhibited in ASC-deficient microglia. We also demonstrated that ASC inflammasome activation was dependent on NLRP3 activation, using the well-characterized NLRP3 inhibitor MCC950. Furthermore, we demonstrated that Tau seeds are taken up into lysosomes and that subsequent inflammasome activation is dependent on cathepsin B activity, a known NLRP3–ASC inflammasome activator. We herewith demonstrate a similar mechanism of inflammasome activation by Tau seeds as previously demonstrated for aggregated Aβ [38]. Previous studies have indicated that Tau is readily taken up by microglial cells in vitro and in vivo [2, 8], which is in line with our current findings demonstrating NLRP3–ASC inflammasome activation following Tau seed uptake in microglia. We furthermore confirmed the presence of punctated Tau staining in microglia in Tau transgenic mice, and we demonstrated the presence of punctated ASC and NLRP3 staining in microglia in Tau transgenic mice, indicative for inflammasome activation in Tau transgenic mice in vivo.

### NLRP3–ASC inflammasome activation, a key regulator of IL-1β signaling, exacerbates Tau pathology

The strong neuroinflammatory and more specifically strong microgliosis component in Tauopathies raises questions about its role in the disease process. Does microglial activation act as a mere bystander in the pathogenetic process of Tauopathies or does it actively contribute to the pathogenetic process, a question which is intensively pursued in the field. Accumulating evidence supports the latter hypothesis, thereby rendering neuroinflammation a therapeutic target. This includes accumulating studies elegantly indicating a contributory role of IL-1β signaling to Tau pathology. IL-1β signaling was shown to exacerbate Tau phosphorylation and Tau pathology in a variety of in vivo models, using different experimental paradigms including genetic models, anti-IL-1β antibodies, IL-1β receptor agonists, among others [7, 14, 29, 58, 63, 65]. Using different experimental approaches these strongly complementary studies demonstrated an exacerbating role of IL-1β on Tau pathology [7, 14, 29, 58, 63, 65]. This is in line with our current findings, demonstrating a beneficial effect of inhibition of NLRP3–ASC inflammasome activation, which is a crucial regulator of IL-1β secretion. Inhibition of IL-1β release in the absence of NLRP3–ASC activation may contribute to decreased Tau pathology observed in our model. Furthermore, Yoshiyama et al. previously demonstrated that microgliosis precedes Tau pathology and neurodegeneration in PS19 mice, using immunostaining and PET ligand studies [101]. We here confirm these findings in an independent way showing that microglial activation precedes Tau pathology in this model. The authors also demonstrated that Tau pathology and Tau-induced neurodegeneration could be prevented by immunosuppression [101]. Together, these data strongly indicate that microgliosis and IL-1β contribute to the pathogenetic neurodegenerative process and Tau pathology. In this work, we demonstrated that NLRP3–ASC inflammasome activation exacerbated exogenously and non-exogenously seeded Tau pathology in vivo. In view of the role of NLRP3–ASC in cleavage of pro-IL-1β to active IL-1β, our findings are in line with a crucial role of IL-1β signaling in Tau pathology, but extend beyond previous data, by demonstrating the implication of the NLRP3–ASC inflammasome and Tau seeding.

### NLRP3–ASC inflammasome exacerbates prion-like or templated Tau-seed induced Tau pathology

We here demonstrated that ASC inflammasome actively contributes to prion-like seeding of Tau pathology. Prion-like or templated seeding of Tau pathology has been reproducibly and consistently recapitulated in vitro and in vivo [24–26, 30, 32, 33, 36, 37, 44, 49, 50, 56, 77, 84, 89, 96, 97]. Tau seeds have been demonstrated to be present in brains of transgenic mice and brains of AD patients and patients with Tauopathies [26, 45]. This strongly underscores the importance and relevance of this mechanism. Hence, prion-like propagation of Tau pathology is generally considered as a compelling mechanism to contribute to the spatio-temporal progression of Tau pathology in brains of AD patients [9] and Tauopathy patients. As progression of Tau pathology strongly correlates with progression of the symptoms in AD patients, its inhibition is considered as a prime therapeutic target [9, 86]. We here demonstrate that exogenous Tau seeding resulted in decreased Tau-seed induced pathology in ASC-deficient mice compared to mice expressing ASC, highlighting an exacerbating role of ASC inflammasome activation in exogenously seeded Tau pathology. Previous studies have demonstrated that microglial elimination decreased prion-like propagation of Tau pathology in an AAV-based model of templated seeding [2] and demonstrated that microglial activation aggravated Tau-seeded Tau pathology [68]. Our findings are in line with these previous reports highlighting an active role of microglia in Tau-seeded Tau pathology [2, 68]. Here we identify ASC-dependent inflammasome as modulator of templated Tau-seeded Tau pathology, using a well-validated model of templated seeding of Tau pathology. Our findings further strengthen a contributory role of microglia in prion-like propagation of Tau pathology, which opens interesting avenues for further research and translational potential.

While we here demonstrated a modulatory role of NLRP3–ASC inflammasome in transgenic mice in vivo, it remains to be considered whether conditions in AD patient brains are compatible with Tau-seed induced inflammasome activation. Factors or requirements to be considered include (i) presence of Tau seeds and concentrations of Tau seeds in AD/Tauopathy brains, (ii) extracellular localization of Tau seeds and (iii) priming of microglia for subsequent Tau-seed induced inflammasome activation. Importantly, our current data demonstrate that ASC inflammasome contributes to exogenously and non-exogenously seeded Tau pathology in Tau mice in vivo. Hence, our experimental data indicate that Tau seed concentrations in Tau transgenic mice and their localization—most parsimoniously extracellular—are compatible with microglial inflammasome activation in this model. As Tau seeds have been demonstrated to be present in brains of AD/Tauopathy patients [26, 45] using a similar assay as in Tau transgenic mice, we anticipate that in brains of patients Tau seeds may activate ASC inflammasome and contribute to Tau-seeded Tau pathology. While activation of NLRP3–ASC inflammasome in primary Tauopathy patients remains to be demonstrated, it must be noted that Tau has been detected in microglia, in Tauopathy patients [8], and IL-1β levels were increased in brains of Tauopathy patients [5, 8, 42]. It furthermore needs to be considered, based on our in vitro data, that priming may be important for subsequent ASC-dependent inflammasome activation by Tau seeds. Priming of microglia, in AD brains, can obviously occur by amyloid pathology. However, in primary Tauopathies, chronic pathological changes in Tau, may first prime microglia, rendering them subsequently susceptible to Tau-seed induced ASC inflammasome activation. As we here demonstrated that ASC inflammasome contributed to Tau-seeded pathology in vivo—in the absence of amyloid pathology—we show that Tau pathology per se is sufficient to activate ASC inflammasome in vivo. This could be explained by the fact that in vivo inflammasome activation does not require priming, or by the fact that pathological changes in Tau already may prime microglia rendering them subsequently susceptible to inflammasome activation.

Our experimental data provide the proof that pathological relevant concentrations of Tau seeds observed in our models are sufficient to drive inflammasome activation, probably through an extracellular phase, and following a preceding priming phase, which is exerted by early pathological Tau changes or by different DAMPs—including Aβ. These conditions are anticipated to be present also in brains of AD/Tauopathy patients, as Tau seeds have been detected in brains of AD patients, similar as demonstrated for Tau transgenic mouse brains, while further research is required. However, Tau was detected in microglia in Tauopathy brains, and IL-1β has been shown to be increased not only in brains of AD patients, but also in Tauopathy patients [5, 8, 42], in line with inflammasome activation in patients.

Taken together, we here identified that NLRP3–ASC inflammasome activation contributes to Tau-seeded Tau pathology in in vivo models, opening interesting avenues for further research and translational potential.

### Mechanisms contributing to the exacerbating effect of NLRP3–ASC inflammasome activation on Tau pathology

The underlying mechanism of decreased Tau-seed induced Tau pathology in ASC-deficient mice may relate to several factors and mechanisms, most probably acting in a combined fashion. First, (i) ASC–NLRP3 inflammasome deficiency was previously shown to have beneficial effects on Aβ amyloid pathology by skewing microglial phenotypes to a more beneficial microglial phenotype [42]. This microglial phenotype exerts more protective functions by increasing phagocytosis and breakdown of aggregated Aβ or aggregated proteins and by secretion of different cytokine and chemokine profiles [40–42]. Similar mechanisms may be involved in the modulatory effect on Tau pathology. In addition, (ii) ASC deficiency inhibits IL-1β secretion [71], which has been previously shown to alter Tau as outlined above, more particularly IL-1β signaling increases Tau phosphorylation and generates Tau forms more prone to aggregate [7, 14, 29, 58, 63, 65]. Furthermore, (iii) a role for microglia in secretion of exosomes containing Tau seeds has also been demonstrated. Finally, and interestingly, (iv) it was recently shown that ASC specks, which are aggregated ASC forms, can be released and induce ASC inflammasome activation by a ‘prion-like’ mechanism [4, 10, 23]. Notably, ASC specks were shown to contribute to Aβ amyloid pathology, by cross-seeding Aβ aggregation [10, 23, 95]. Cross-seeding between different forms of aggregating proteins is being increasingly recognized as a contributing mechanism in neurodegenerative diseases, which are mostly characterized by mixed pathologies, i.e., aggregating proteins characteristic of different neurodegenerative disorders. We anticipate that a combination of these mechanisms contributes to the inhibitory effect of ASC deficiency on Tau-seed induced Tau pathology demonstrated in this work. It is not clear whether it will be possible to pinpoint the respective contributions of these mechanisms, but follow-up of our current work is anticipated, including single-cell sequencing to identify specific populations of microglial cells and analysis of cross-seeding potential of ASC specks to induce Tau pathology, extending beyond the scope of the current paper.

### NLRP3 inflammasome as therapeutic target extended to AD and Tauopathies, concomitantly affecting three crucial processes in AD

Following the identification of the role of ASC-dependent inflammasome in exacerbating Tau-seeded Tau pathology, we here demonstrated that pharmacological inhibition of NLRP3 using the well-characterized NLRP3 inhibitor MCC950 prevented Tau-seed induced Tau pathology in a dose-dependent way. NLRP3 is increasingly scrutinized as therapeutic target for a variety of diseases. NLRP3 inhibition is intensively investigated, as therapeutic target for infectious diseases, autoimmune diseases and autoinflammatory diseases [59–61, 66, 93]. Recently, IL-1β blocking therapy was demonstrated to decrease the risk for stroke in coronary artery disease patients with inflammatory atherosclerosis [82], while its modulatory role in cancer [83], macular degeneration [91] and neurodegenerative disorders [1, 38, 40–42, 95] is also intensively investigated [93]. Hence, our data not only highlight the implication of the NLRP3–ASC axis in Tau-seeded Tau pathology, but also highlight the therapeutic potential of NLRP3 inhibitors to target Tau-seeded Tau pathology. Interestingly, previous data very elegantly identified NLRP3–ASC inflammasome as a modulator of beta-amyloid pathology: Aβ aggregates activate NLRP3–ASC inflammasome [38], NLRP3–ASC inflammasome deficiency inhibits amyloid pathology [42] and ASC specks induce templated cross-seeding of amyloid pathology [95] contributing to spreading of amyloid pathology in vivo. Hence, combined, our results identify NLRP3–ASC as a therapeutic target which concomitantly affects three key processes in AD, including besides Tau and prion-like seeded Tau pathology also Aβ and neuroinflammation. In the search for multi-target therapy, the NLRP3–ASC axis hence shows interesting potential.

## Conclusion

Taken together, we have here demonstrated a novel mechanism of Tau-seed induced microglial changes, by activation of the NLRP3–ASC axis. We furthermore demonstrate that inhibition of the NLRP3–ASC axis inhibits Tau-seeded Tau pathology as well as non-exogenously seeded Tau pathology. Our findings hereby demonstrate a very strong parallel between Aβ and Tau in activating the inflammasome and microglia. It was previously shown by Halle et al. that Aβ activates the NLRP3 inflammasome. These findings were further extended to demonstrate beneficial effects of inhibition of NLRP3 inflammasome on development of amyloid pathology. Recently, the potential of cross-seeding of Aβ with ASC specks was identified. Taken together, we here provide a novel means to interfere with Tau-seeded Tau pathology. The NLRP3–ASC inflammasome, which is explored for therapeutic strategies for a variety of diseases hence presents as an interesting therapeutic approach targeting key pathogenetic processes in AD, including Tau pathology besides Aβ and neuroinflammation.

## Electronic supplementary material

Below is the link to the electronic supplementary material.

Supplementary material 1 (TIFF 38768 kb)

Supplementary material 2 (TIFF 85497 kb)

Supplementary material 3 (TIFF 14679 kb)

Supplementary material 4 (TIFF 42576 kb)

Supplementary material 5 (PDF 508 kb)

Supplementary material 6 (PDF 572 kb)
