# Supplementary figures and images for: Aggregated Tau activates NLRP3–ASC inflammasome exacerbating exogenously seeded and non-exogenously seeded Tau pathology in vivo
Source: Acta Neuropathol. 2019 Feb 5;137(4):599–617. doi: 10.1007/s00401-018-01957-y (PMC6426830; doi:10.1007/s00401-018-01957-y)

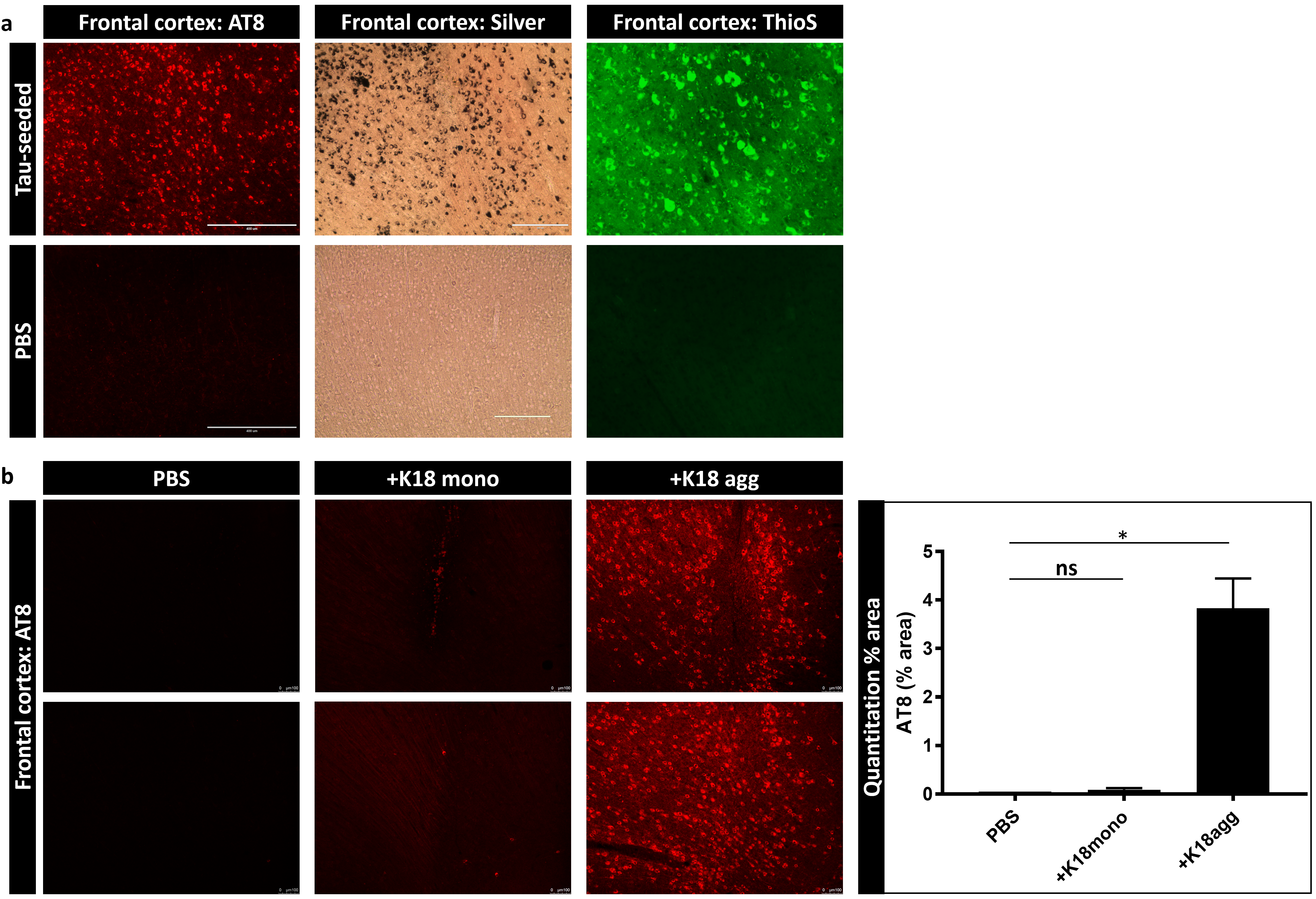

Supplement: Supplementary file 1 — Supplementary material 1 (TIFF 38768 kb) [file 401_2018_1957_MOESM1_ESM.tif]

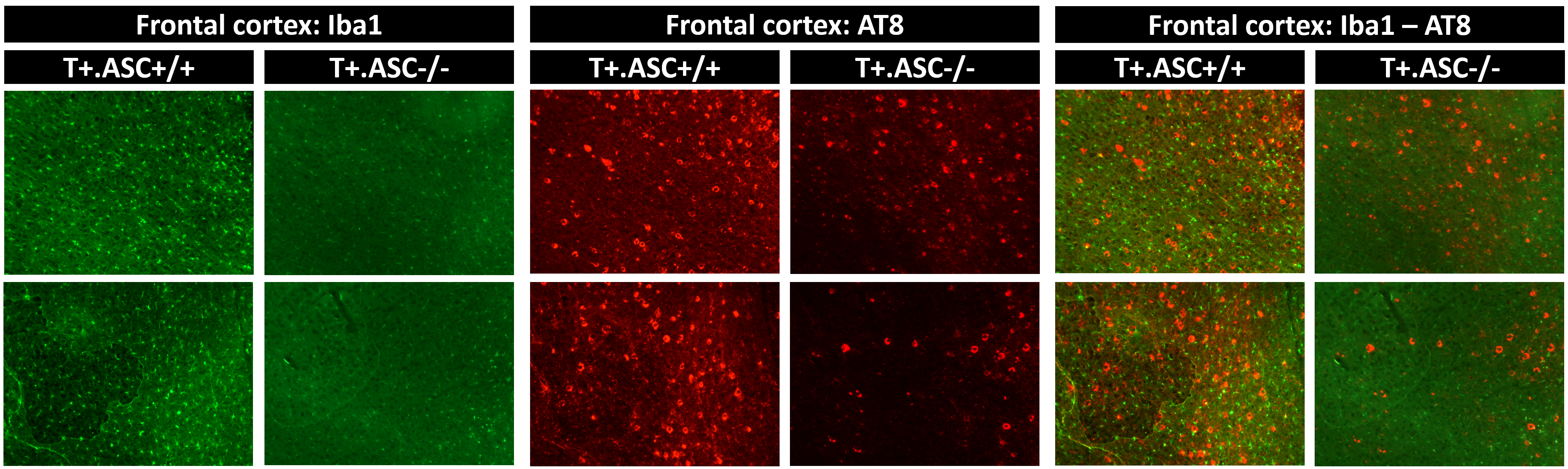

Supplement: Supplementary file 3 — Supplementary material 3 (TIFF 14679 kb) [file 401_2018_1957_MOESM3_ESM.tif]

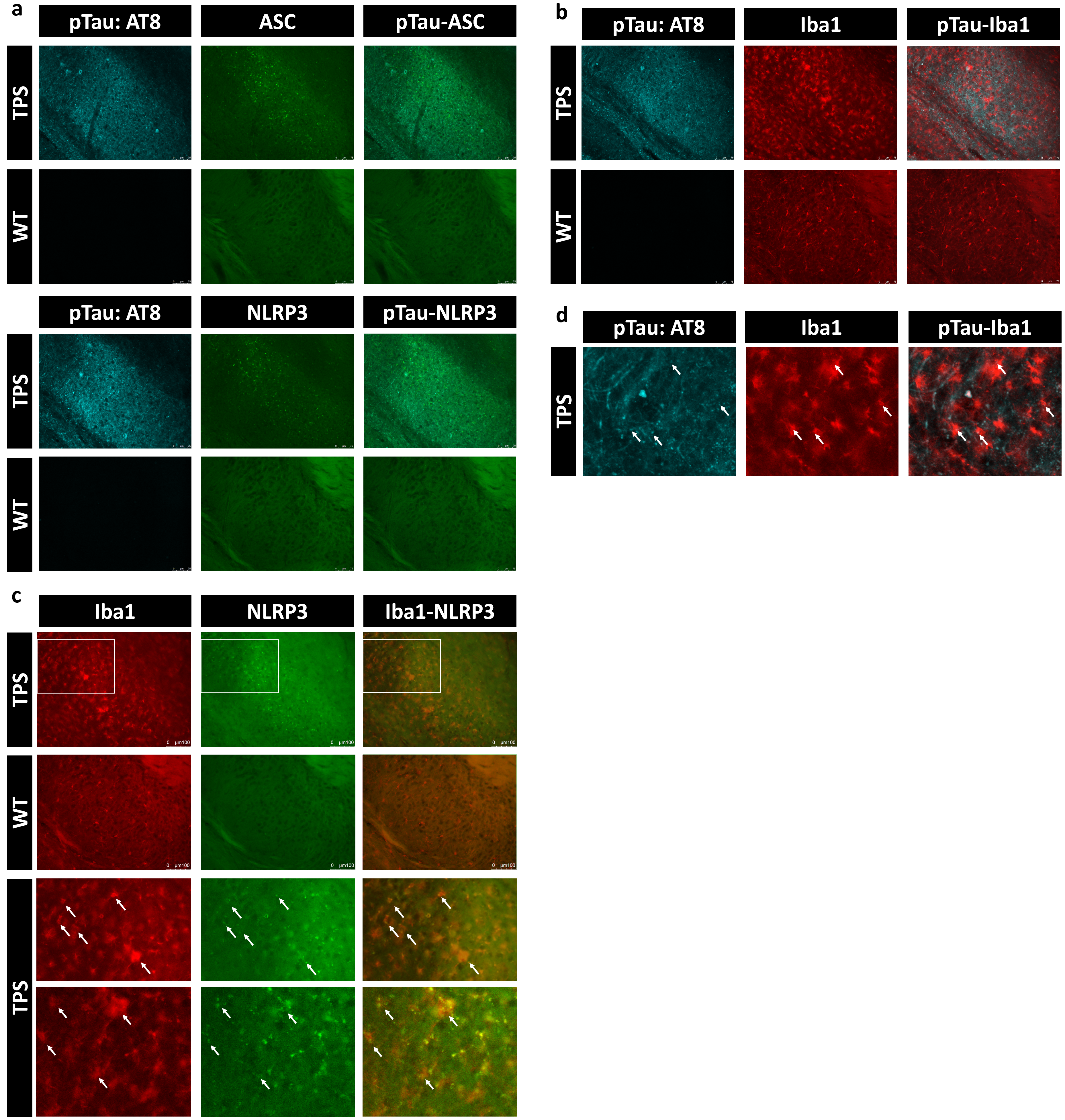

Supplement: Supplementary file 4 — Supplementary material 4 (TIFF 42576 kb) [file 401_2018_1957_MOESM4_ESM.tif]
