# Supplementary material for: Aggregated Tau activates NLRP3–ASC inflammasome exacerbating exogenously seeded and non-exogenously seeded Tau pathology in vivo
Source: Acta Neuropathol. 2019 Feb 5;137(4):599–617. doi: 10.1007/s00401-018-01957-y (PMC6426830; doi:10.1007/s00401-018-01957-y)
Supplement: Supplementary file 5 — Supplementary material 5 (PDF 508 kb) [file 401_2018_1957_MOESM5_ESM.pdf]

Aggregated Tau activates NLRP3-ASC inflammasome exacerbating exogenously seeded and non-exogenously seeded Tau pathology in vivo

Acta Neuropathologica

Ilie-Cosmin Stancu[1,2], Niels Cremers[1], Hannah Vanrusselt[1], Julien Couturier[2], Alexandre Vanoosthuyse[2], Sofie Kessels[1], Chritica Lodder[1], Bert Brône[1], François Huaux[3], Jean-Noël Octave[2], Dick Terwel[1], Ilse Dewachter[1,2]

Affiliations:

1. Biomedical Research Institute, Hasselt University, 3500 Hasselt, Belgium
2. Institute of Neuroscience, Alzheimer Dementia Group, Catholic University of Louvain, 1200 Brussels, Belgium
3. Louvain Center for Toxicology and Applied Pharmacology, Catholic University of Louvain, 1200 Brussels, Belgium

Corresponding author: Ilse Dewachter, ilse.dewachter@uhasselt.be, phone: +32 11286957

Supplemental Figure legends

Online Resource 1 Tau-seeding induced Tau-pathology in Tau transgenic mice

a Injections of Tau-seeds (upper panel) or PBS (lower panel) were performed in Tau transgenic mice at the age of 3 months and analyzed 6 months post-injection. Representative images of the frontal cortex at the site of injection, stained with anti-P-Tau (AT8; red) antibody shows robust induction of Tau-pathology in Tau-seeded, absent in age-matched PBS injected, Tau transgenic mice. Silver and ThioS staining demonstrate the presence of mature NFTs following injection of Tau seeds in Tau transgenic mice. **b** Comparison of injection of pre-aggregated Tau seeds (K18 agg), non-aggregated monomeric Tau (K18 mono) and PBS in frontal cortex of 3 months old Tau transgenic mice. Representative images of frontal cortex at the injection site, stained with anti-P-Tau (AT8; red) antibody, shows robust induction of Tau-pathology following injection of pre-aggregated Tau seeds (K18 agg), nearly absent following injection of non-aggregated monomeric Tau (K18 mono), and absent in age-matched PBS injected Tau transgenic mice. Quantitative analysis demonstrates significant induction of Tau-pathology following injection of pre-aggregated Tau compared to PBS injected Tau mice only (n=3, PBS; n=4, $Tau_{k18mono}$; n=4, Tau_{k18agg}; Kruskall-Wallis ANOVA with Dunn's multiple comparison post hoc, *p<0,05).

Online Resource 2 ASC deficiency reduces induction of Tau pathology following Tau-seeding in Tau transgenic mice (complementary to Figure 2)

a Immunohistological analysis of Tau-seed induced Tau pathology in brains of T+. ASC+/+ and T+.ASC-/- mice. Tau seeding was performed at 6 months of age and analyzed 3 months post-injection. Representative images of anti-P-Tau (AT8; red) staining in frontal cortex of T+. ASC+/+ and T+.ASC-/- mice are presented. Quantitative analysis of the area stained with AT8 reveals a significantly lower induction of Tau pathology in T+.ASC-/- compared to T+.ASC+/+ mice (n=6, T+.ASC+/+; n=7, T+.ASC-/-; Mann-Whitney t-test, *p<0,05). Silver (black) and This (green) staining demonstrate the presence of mature NFTs following Tau seeding in Tau transgenic mice, representative images for each staining of frontal cortex are shown. Tau-seeding induced Tau pathology (NFTs) assessed by silver and ThioS staining was significantly less in T+.ASC-/- compared to T+.ASC+/+ mice (n=6, T+.ASC+/+; n=7, T+.ASC-/-; Mann-Whitney t-test, *p<0,05). These results were obtained in a first cohort (cohort 1) used to evaluate the potential implication of inflammasome activation in Tau-seeded Tau pathology. The study was subsequently extended with a new cohort of mice presented in figure 2 of the manuscript. The implication of ASC in Tau-seeded Tau pathology has been identified in the 2 different cohorts analyzed independently. **b** Propagation of Tau pathology was assessed by analyzing Tau-seed induced Tau pathology at the contralateral side in brains of T+. ASC+/+ and T+.ASC-/- mice. Representative images of AT8 (red) staining in frontal cortex of the contralateral side in T+.ASC+/+ and T+.ASC-/- mice (data presented in figure 2) are presented. Quantitative analysis of the area stained with AT8 reveals a significantly lower induction of Tau pathology in T+.ASC-/- compared to T+.ASC+/+ mice (n=6, T+.ASC+/+; n=5, T+.ASC-/-; Mann-Whitney t-test, *p<0,05). **c** Biochemical analysis was performed on sarkosyl insoluble Tau fractions obtained from the frontal cortices of T+.ASC+/+ and T+.ASC-/- mice following Tau-seeding in frontal cortex. Representative samples of sarkosyl insoluble Tau following anti-P-Tau (AT8) immunoblotting are shown. Western blotting using AT8 on sarkosyl insoluble

fractions extracted from T+. ASC+/+ and T+.ASC-/- mice, revealed a significant decrease in sarkosyl insoluble Tau in T+.ASC-/- compared to T+. ASC+/+ mice following Tau-seeding in frontal cortex (n=5, T+.ASC+/+; n=4, T+.ASC-/-; Mann-Whitney t-test, *p<0,05).

Online Resource 3 Tau seeding induced microgliosis is decreased in ASC deficient Tau mice

Representative images of Iba1 (green) staining in brains of Tau-seeded T+.ASC+/+ compared to T+.ASC-/- are presented demonstrating increased microgliosis in Tau-seeded T+.ASC+/+ mice compared to T+.ASC-/- mice.

Online Resource 4 Uptake of Tau seeds in microglia and inflammasome activation in brains of Tau transgenic mice

a Immunofluorescent staining was performed on brain sections of aged-matched Tau transgenic (TPS) and wild-type (WT) mice. Co-immunostaining of anti-P-Tau (AT8; blue) and anti-ASC (green) or anti-NLRP3 (green) antibody, revealed increased and punctated staining in the brains of Tau transgenic mice, not detected in non-transgenic mice. Punctated staining is indicative for ASC and NLRP3 aggregate formation, supporting induction of inflammasome activation in brains of Tau transgenic mice. **b** Immunostaining of anti-P-Tau (AT8; blue) and anti-Iba1 (red) revealed microglial activation in the brains of Tau transgenic (TPS) mice not detected in non-transgenic (WT) mice. **c** Higher magnification of the staining presented in the upper panel, revealed the presence of NLRP3-positive punctae (green) within microglia (Iba1; red), in the brain of Tau transgenic (TPS) mice. **d** Higher magnification of the immunostaining presented in panel b, revealed the presence of anti-P-Tau (AT8; blue) staining within microglia (Iba1; red), indicating the uptake of Tau forms into the microglia, in the brain of Tau transgenic (TauP301S) mice.
