# Supplementary material for: Aggregated Tau activates NLRP3–ASC inflammasome exacerbating exogenously seeded and non-exogenously seeded Tau pathology in vivo
Source: Acta Neuropathol. 2019 Feb 5;137(4):599–617. doi: 10.1007/s00401-018-01957-y (PMC6426830; doi:10.1007/s00401-018-01957-y)
Supplement: Supplementary file 6 — Supplementary material 6 (PDF 572 kb) [file 401_2018_1957_MOESM6_ESM.pdf]

Aggregated Tau activates NLRP3-ASC inflammasome exacerbating exogenously seeded and non-exogenously seeded Tau pathology in vivo

Acta Neuropathologica

Ilie-Cosmin Stancu[1,2], Niels Cremers[1], Hannah Vanrusselt[1], Julien Couturier[2], Alexandre Vanoosthuyse[2], Sofie Kessels[1], Chritica Lodder[1], Bert Brône[1], François Huaux[3], Jean-Noël Octave[2], Dick Terwel[1], Ilse Dewachter[1,2]

Affiliations:

1. Biomedical Research Institute, Hasselt University, 3500 Hasselt, Belgium
2. Institute of Neuroscience, Alzheimer Dementia Group, Catholic University of Louvain, 1200 Brussels, Belgium
3. Louvain Center for Toxicology and Applied Pharmacology, Catholic University of Louvain, 1200 Brussels, Belgium

Corresponding author: Ilse Dewachter, ilse.dewachter@uhasselt.be, phone: +32 11286957

Supplemental Materials and Methods

Monomeric and aggregated Tau seeding in vivo

To analyze the potency of monomeric and aggregated Tau (Tau-seeds) seeding on Tau pathology, we performed injection of pre-formed Tau fibrils and monomeric Tau in TauP301S (PS19) mice. The mice were deeply anaesthetized by intraperitoneal injection of Ketamine/Xylazine mixture (Ketalar/Rompun) and placed in the stereotaxic apparatus (Kopf Instruments). Stereotactic injections of pre-aggregated Tau (K18 agg), monomeric Tau (K18 mono) and phosphate-buffered saline (PBS) were performed as described previously [2]. Briefly, sonicated Tau-seeds (K18 agg; 5 µl; 333 µM), sonicated monomeric Tau (K18 mono; 5 µl; 333 µM) and PBS (5 µl) were injected using a 10 µl Hamilton syringe in the frontal cortex (A/P, +2.0; L, +1.4; D/V, −1.0; relative to bregma) at a rate of 1 µl/minute. After injection, the needle was kept in place for additional 5 min before gentle withdrawal. The injected mice were sacrificed at the indicated time post-injection for immunohistochemical and biochemical analysis.

Immunohistochemical analysis

Immunohistological analysis was performed as described previously [1-3]. Briefly, the brains were dissected, after 2 min of transcardial perfusion with ice cold PBS (Sigma-Aldrich, St. Louis, USA) and fixed for 24 h in 4% PFA-PBS at 4°C. Sagittal sections (40µm) of mice brains were generated with a vibrating HM650V microtome (Thermo Fisher Scientific, Waltham, MA, USA) and were preserved in PBS + 0.1 % sodium azide. The free-floating sagittal brain sections were first washed twice in PBS and then three times in PBS + 0.1% TritonX-100 (PBST). Permeabilization of the tissue was performed using PBST + methanol (1:1) for 10 min, and subsequently blocked with PBST + 5% milk. The following primary antibodies were used: anti-Tau P-S202/T205 (AT8, 1:100; Thermo Fisher Scientific) for Tau-pathology, anti-Iba1 (Iba1, 1:500; Wako-Chemical GmbH, DE) for microglia, and anti-ASC (AL177, 1:500; Adipogen Life Sciences) and anti-NLRP3 (1:200; Adipogen Life Sciences) for ASC and NLRP3 proteins, respectively. The primary antibodies were incubated for 2 hours at room temperature (RT) or overnight at 4°C, followed by appropriate Alexa-coupled secondary antibody (1:500) in PBST + 5% milk for 1h at RT. The brain slices were finally washed three times with PBST then twice with PBS and mounted with Fluoroprep mounting medium (BioMérieux, Marcy-l'Etoile, France). Image acquisition was performed with a Leica DM450B fluorescence microscope (Leica, Wetzlar, DE), EVOS FL Auto Imaging System (Thermo Fisher Scientific) and standard light microscope. Image analysis was performed with Image J (National Institutes of Health) blinded to the genotype and/or treatment of the mice. Briefly, the fluorescent TIFF images were converted to 16-bit images and then thresholded using the Image J Default method to allow quantification of the stained area without detection of the background staining, the threshold was then applied to all sections. For quantification of the silver staining area, the 16-bit images were inverted prior to the same thresholding procedure as above. The amount of staining was measured as the percentage of area occupied by positive signal within the brain region (frontal cortex, hippocampal CA1). The results were statistically processed with GraphPad Prism 7.04 software (GraphPad, San Diego, CA, USA).

Biochemical analysis of insoluble Tau

For immunoblotting analysis, the cortex was dissected and snap-frozen in liquid nitrogen. Total homogenates, sarkosyl soluble and sarkosyl insoluble fractions were prepared as

described previously [2]. Briefly, the cortex was homogenized with 20 strokes at 700 rpm on ice, in six volumes of Tris-NaCl buffer (25 mM TrisHCl, 150 mM NaCl, 1 mM EDTA, 1 mM EGTA, 5 mM $Na_4P_2O_7$, 30 mM NaF, pH 7.6) with addition of 1 mM PMSF, EDTA-free protease and phosphatase inhibitors cocktail (F. Hoffmann-La Roche AG, Basel, CH). Half of this total homogenate was stored at -80°C and half was used for the sequential extraction, by ultracentrifugation at 150,000 g for 30 min at 4°C. The pellet was resuspended with equal volume of 10 mM TrisHCl, 0.8 M NaCl, 10 % sucrose, pH 7.6 with addition of 1 mM PMSF, EDTA-free protease and phosphatase inhibitors cocktail. Subsequently it was centrifuged at 20,000 g for 30 min at 4°C, the supernatant was incubated with 1 % sarkosyl (N-lauroylsarcosine sodium salt; Sigma-Aldrich) for 1 h at RT followed by ultracentrifugation at 150,000 g for 30 min at 4°C. The supernatant (sarkosyl soluble fraction) and the resuspended pellet (sarkosyl insoluble fraction) were stored at -80°C. Proteins were quantified using BCA Protein Assay kit (Thermo Fisher Scientific, Waltham, MA, USA). For immunoblotting equal amounts of proteins were loaded on 8% Tris-glycine gels (Invitrogen, Life Technologies, Carlsbad, CA, USA). Immunoblotting was performed with anti-Tau P-S202/T205 (AT8, 1/1000; Thermo Fisher Scientific, Waltham, MA, USA) and developed using Pierce ECL Plus Substrate kit (Thermo Fisher Scientific). Immunoblots were imaged using an ImageQuant LAS 4000 imaging system (GE Healthcare Life Sciences, UK) and relative protein amounts were quantified from the immunoblots using Gel analysis method of Image J (National Institutes of Health).

References

1 Peeraer E, Bottelbergs A, Van Kolen K, Stancu IC, Vasconcelos B, Mahieu M et al (2015) Intracerebral injection of preformed synthetic tau fibrils initiates widespread tauopathy and neuronal loss in the brains of tau transgenic mice. Neurobiol Dis 73: 83-95 Doi 10.1016/j.nbd.2014.08.032

2 Stancu IC, Vasconcelos B, Ris L, Wang P, Villers A, Peeraer E et al (2015) Templated misfolding of Tau by prion-like seeding along neuronal connections impairs neuronal network function and associated behavioral outcomes in Tau transgenic mice. Acta Neuropathol 129: 875-894 Doi 10.1007/s00401-015-1413-4

3 Vasconcelos B, Stancu IC, Buist A, Bird M, Wang P, Vanoosthuyse A et al (2016) Heterotypic seeding of Tau fibrillization by pre-aggregated Abeta provides potent seeds for prion-like seeding and propagation of Tau-pathology in vivo. Acta Neuropathol 131: 549-569 Doi 10.1007/s00401-015-1525-x
